# Postoperative adhesion formation: the role of peritoneal macrophages and targeting therapy

**DOI:** 10.3389/fimmu.2025.1601642

**Published:** 2025-09-02

**Authors:** Zhong-Yuan Liu, Ren-Feng Li, Hong-Yan Qin, Peng-Fei Ma

**Affiliations:** ^1^ Department of Hepatobiliary and Pancreatic Surgery, The First Affiliated Hospital of Zhengzhou University, Zhengzhou, China; ^2^ Department of Medical Genetics and Development Biology, Air Force Medical University, Xi’an, China

**Keywords:** postoperative adhesion, peritoneal macrophages, molecular mechanisms, serosal repair, adhesion prevention within the peritoneal cavity, peritoneal macrophage subpopulations

## Abstract

**Background:**

Postoperative adhesion (PA) is the most common complication of abdominal or pelvic surgery, resulting in various complications, including small bowel obstruction, secondary female infertility, chronic abdominal pain and even death. Unfortunately, there are few effective methods for the prevention and treatment of it. Previous studies confirmed that coagulation response, inflammation, fibrosis and fibrinolysis played a central role in PA formation and a variety of cells, such as monocytes, macrophages, fibroblasts, neutrophils and mesothelial cells participated in this process. Peritoneal macrophages are prominent immune cells that maintain homeostasis and coordinate cavity wound healing in serosal cavities.

**Aim of review:**

In this review, we analyze the role of peritoneal macrophages in PA formation. it also presents the latest treatment and prevention strategies targeting peritoneal macrophages. This review focuses to provide an immunological theoretical basis and new target for the prevention and treatment of PA.

**Key scientific concepts of review:**

Peritoneal macrophages recognize sterile or infected injury, initiate repair, form aggregates, and regulate coagulation, inflammation, fibrosis and fibrinolysis in PA formation. Therefore, in the most of existing strategies to prevent PA formation, the biological mechanism is related to the function of peritoneal macrophages. Targeting macrophages to prevent PA formation holds great promise.

## Graphical Abstract

Schematic diagrams of some mechanisms regarding the roles of peritoneal macrophages in coordinating the coagulation, inflammation, fibrosis and fibrinolysis and MMT in postoperative adhesion formation. **(A)** Mesothelium injury induces acute inflammatory response resulting in the exudation of fibrinogen from vessels. Meanwhile, activated coagulation factors, such as FV, FX, companying with Ca^2+^ and phospholipids (PL) activates prothrombin into thrombin, which is able to cleave fibrinogen and promote the formation of fibrinous colts. Subsequently, plasminogen activators, such as tPA and uPA, activate plasminogen and initiate fibrinolysis response incurring fibrin clots degradation. **(B)** Heterogenous peritoneal macrophages play a key role in the formation of PA. First, they release lots of inflammatory cytokines, such as TNF-α, IL-6, IL-12, IL-1β and chemokines, such as CCL5, CXCL9, CXCL10, leading to tissue injury and immune cells aggregations. Second, as the main source of peritoneal coagulation factors, such as FV, FVII and FX, peritoneal macrophages are necessary for peritoneal coagulation response. In the end, peritoneal macrophages can activate mesothelial cells by secreting TGF- β, IL-10 or directly touching with them with multiple mechanisms, such as CX3CR1-CX3CL1. (Dotted line means this phenotype is not confirmed in the model of peritoneal adhesion, but has been proved in other peritoneal models, such as peritoneal dialysis model.).

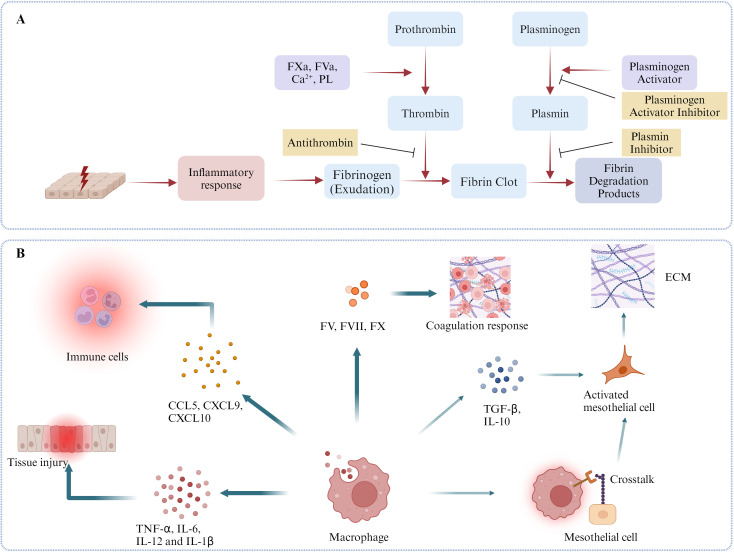

## Highlights

- Peritoneal macrophages play a crucial role in the formation of peritoneal adhesion.
- Peritoneal macrophages are the main immune cells in peritoneal cavity.
- Peritoneal macrophages recognize sterile or infected injury, initiate repair, form aggregates, and regulate coagulation, inflammation, fibrosis and fibrinolysis in PA formation.
- Targeting peritoneal macrophages have great potential to prevent and treat peritoneal adhesion.

## 1 Introduction

Postoperative adhesions are pathological fibrotic bands that form permanent scar tissue between internal organ surfaces, the omentum, and abdominal wall structures following surgical intervention (1–3). It is estimated that more than 90% abdominal or pelvic surgeries will lead to the formation of PA and cause small bowel obstruction, secondary female infertility, chronic abdominal pain and even death (4–6). Despite substantial investments of time and resources in developing anti-adhesion strategies - including surgical technique refinements, physical barriers, and chemical agents - no currently available method provides fully satisfactory prevention of peritoneal adhesion formation (7). Furthermore, once adhesions formed, surgery is the only effective way to lyse them, which predisposes the patients to further adhesion (8, 9). The Surgical and Clinical Adhesions Research group has reported that 5.7% of readmissions are directly related to adhesions, nearly a quarter of which occur in the first postoperative year (10). Thus, there is an urgent need to develop effective ways to minimize PAs.

PA is mainly mediated by four factors: inflammatory pathways, coagulation responses, fibrosis and fibrinolysis (2). Other factors, such as hypoxia, infections also have impacts on PA. When tissue damage occurs, hemostasis or coagulation is the initial process that happens to stop blood loss (11). Ample thrombinogen is activated into thrombin in the presence of activated factor V and X, Ca^2+^ and a phospholipid surface. Simultaneously, tissue injury, foreign body, blood or bacteria can trigger the inflammatory response (12). Subsequently, a large number of inflammatory cells, such as neutrophils, monocytes, macrophages and lymphocytes are recruited to the injury sites to clear pathogens and cellular debris. These immune cells release numerous chemokines, inflammatory cytokines, such as CXCL13 (CXC-chemokine ligand 13), CCR2 (C-C motif chemokine receptor 2), tumor necrosis factor-α (TNF-α), interleukins-1β (IL-1β) and interleukins-6 (IL-6) (13), resulting in more inflammatory cells aggregations and the increased vasopermeability. In the end, abundant fibrinogen exudates out the vascular and are activated by thrombin to contribute to the development of fibrin clots. Furthermore, these inflammatory cells and fibrins promote the formation of mesothelium-bound, fibrin-dependent, multicellular aggregates which exert the functions of controlling the infection and shielding the injury (13, 14). Macrophages identify and clear apoptosis neutrophils via efferocytosis, marking the initiation of the resolution of inflammation (13, 15). During the remission of inflammation, kinds of stromal cell such as fibroblasts, stem cells, migrate into the wound bed and contribute to forming the granulation tissue. Then, these stromal cells are activated by signaling molecules, such as transforming growth factor-β1 (TGF-β1) and vascular endothelial growth factor-α (VEGF-α) (16). Activated fibroblasts present strong proliferation abilities and produce abundant matrix, leading to the generation of new tissue. In addition, some studies have revealed that neutrophils can direct preexisting matrix around the wound to engage in the repair (17). Plasmin, an enzyme used to degrade fibrin, is activated by tissue fibrinogen activator (t-PA) and urokinase fibrinogen activator (u-PA). Plasmin and Matrix metalloproteinases (MMPs) play a vital role in degrading superfluous extracellular matrix (ECM) and tissue remodeling. Eventually, there are two different repair outcomes: complete restoration or fibrotic scar formation (13). Hence, it is crucial to maintain inflammatory responses, coagulation responses, fibrosis, and fibrinolysis in a moderate and balanced pattern.

Peritoneal cavity contains various immune cells which play important roles in the acute inflammatory response given that they are able to phagocytize pathogens, present antigens, and activate other cells. Among these cells, macrophages play a crucial role in modulating the entire process of tissue repair owing to their diverse phenotypes and functional capabilities (9). Within the peritoneal cavity, macrophages serve as the initial sensors of tissue injury, rapidly forming aggregates at the site of damage—similar to platelet aggregation—shortly after injury occurs, resulting in undetectable macrophages in the peritoneal fluid, a phenomenon called macrophage disappearance reaction (MDR) (18, 19). They exhibit multifunctional properties, such as potent phagocytic activity, the ability to secrete a wide array of cytokines to recruit inflammatory cells, and efficient clearance of harmful stimuli, including bacteria (20). Moreover, peritoneal macrophages serve as the important source of coagulation factors (e.g., factors V, VII, and X) in the peritoneal fluid, facilitating rapid hemostasis in emergency situations (21). Furthermore, macrophages secrete TGF-β1, VEGF-α, and MMPs, which may serve as key mediators in mesothelial-to-mesenchymal transition (MMT) and activating collagen-producing, α-smooth muscle actin (α-SMA)-positive myofibroblasts and regulate collagen degradation (12, 22–25). Conversely, studies have demonstrated that complete depletion of peritoneal macrophages leads to impaired tissue repair processes (18). Therefore, peritoneal macrophages play pivotal regulatory roles in wound healing and tissue remodeling. Therapeutic targeting of these macrophages may represent a promising strategy for preventing peritoneal adhesions.

In this review, we highlight recent advances in understanding the immune system’s role - particularly macrophage biology, associated molecular mechanisms and targeting macrophage therapy- in PA formation and targeting macrophage therapy. These insights provide novel perspectives and therapeutic approaches for developing more effective PA prevention strategies.

## 2 Heterogeneous macrophages regulate peritoneal cavity homeostasis

In mammals, there are three main serosal cavities: the peritoneal, pleural, and pericardial cavities. Although they are located in distinct anatomical compartments, they have the same embryological origin, the embryonic coelom, and contain vital organs (26). Among them, the peritoneal cavity is the largest serosal cavity surrounded by two mesothelial layers (visceral and parietal peritoneum) and accommodates the stomach, spleen, intestines, pancreas, reproductive organs, omentum, and so on (27, 28). The peritoneal cavity contains a small volume of peritoneal fluid (5~20 ml in healthy humans, ~50 μl in 6-week-old C57BL/6J mice), which lubricates the surface of visceral tissues, facilitating smooth movement of the visceral and parietal serosa without friction (21, 29). Notably, mouse peritoneal fluid contains remarkably high cellular density, with a concentration of approximately 6.5 × 10^4^ cells/μl - about 20-fold higher than circulating leukocyte levels in peripheral blood (21). What’s more, most leukocytes in the peritoneal cavity are lymphocytes (10-60%) and macrophages (40-60%) (29), and other various immune cells, such as monocytes, dendritic cells, innate lymphoid cells, T cells, B-1 cells, mast cells and natural killer cells are rare (30, 31).

**Figure 1:**
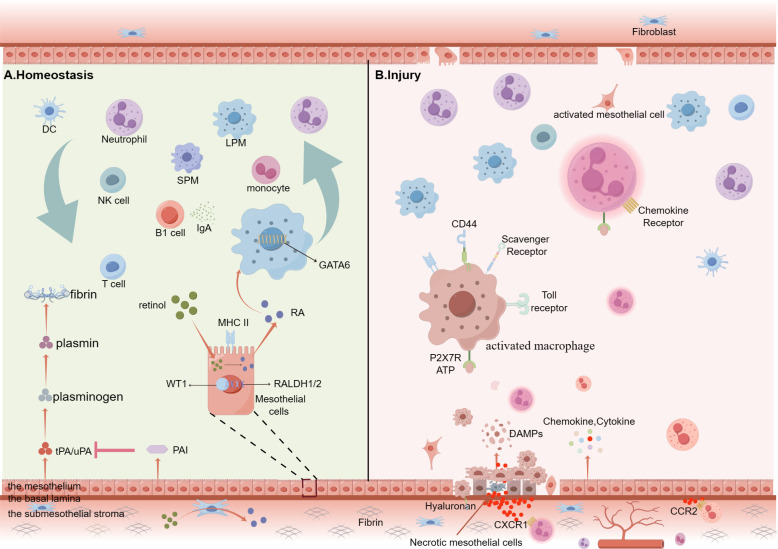
Summary of cellular kinetics of peritoneal immunity cells under homeostasis conditions or on peritoneal sterile injury. **(A)** Peritoneal cavity is surrounded by two mesothelial layers (visceral and parietal peritoneum), and both consisting of three distinctive layers: the mesothelium, a basal lamina and the submesothelial stroma. The mesothelium forms the innermost surface of the peritoneum. Under homeostatic conditions, peritoneal cavity contains abundant free-floating leukocytes, such as lymphocytes (10-60%), macrophages (40-60%), B-1cells, T cells, monocytes, natural killer (NK) cells and so on. Their migration is completely passive because the power is from the fluid shear flow. Mesothelial cells produce both tissue-type, urokinase-type plasminogen activators (tPA and uPA) and the glycoproteins plasminogen activator inhibitors (PAI)-1 and PAI-2. By expressing MHC-II molecules, the mesothelial cells are able to present antigens. RALDH1/2 is the rate-limiting enzyme for retinal metabolism. Mesothelial and fibroblastic stromal cells prompt RALDH1/2 via expressing Wilms’ Tumor 1 (WT1) to produce retinoic acid, which is a pivotal driver of GATA6 expression. B-1 cells secret IgA antibodies which are important ingredient of innate immunity. **(B)** Injury mesothelial cells release DAMPs, such as ATP, which binds to the P2X7 receptors to activate LPMs and ample chemokine and cytokine to recruit abundant immune cells to the injury site. These activated LPMs upregulate the number of scavenger receptors (SRs) and TLRs on the membrane and first attach to the injury mesothelial cells. Mesothelial cells from the wound edge, the opposing surface and distant sites are stimulated to undergo mesothelial–mesenchymal transition (MMT). (By Figdraw.).

Macrophages represent one of the most functionally versatile and phenotypically heterogeneous cell populations, residing in nearly all mammalian tissues where they surveil the local microenvironment and maintain tissue homeostasis. Under physiological conditions, the murine peritoneal cavity harbors at least three distinct macrophage subsets, which are broadly divided into F4/80^hi^ large peritoneal macrophages (LPMs), F4/80^lo^ small peritoneal macrophages (SPMs) and F4/80^int^ intermediate macrophages (32, 33). LPMs represent the resident macrophage population in the peritoneal cavity, exhibiting characteristically larger cell sizes compared to SPMs. In mice, LPMs originate from yolk sac-derived hemogenic endothelium during embryonic development and are subsequently maintained through regulation by tissue-specific niche signals in the peritoneal microenvironment (34–37). SPMs originate from hematopoietic stem cells (HSCs) and demonstrate limited niche occupancy compared to their LPMs counterparts (35). F4/80^int^ macrophages represent a transitional population undergoing differentiation from SPMs to LPMs, as they progressively acquire niche-specific residency and phenotypic characteristics (38). Under steady-state conditions, LPMs are the prominent macrophages, containing approximately 90% of the peritoneal macrophages, whereas SPMs and F4/80^int^ intermediate macrophages rarely found in the healthy peritoneal fluid (39, 40). Peritoneal macrophage subpopulations exhibit distinct turnover kinetics, phenotypic characteristics, and functional properties that correlate with their developmental origins and specific niche environments. These population-specific attributes will be examined in detail in the following section (**Figure 1**).

A recent study identified another three resident macrophage subpopulations sharing a set of core gene signatures in five organs of C57BL/6J mice by using unbiased single-cell RNA sequencing. Based on the expression of the phosphatidylserine receptor T cell immunoglobulin and mucin domain containing 4 (TIMD4), lymphatic vessel endothelial hyaluronan receptor 1 (LYVE1), folate receptor beta (FOLR2)(TLF), major histocompatibility complex II (MHC-II) and CCR2, three resident macrophage subpopulations is defined as MHC-II^hi^ macrophages, CCR2^+^ macrophages and TLF^+^ macrophages (41). Similarly, a study found that two separate macrophage subsets with diverse expressing profiles across lung, heart, dermis and other organs: Lyve1^lo^MHC-II^hi^CX3CR1^hi^ (C-X3-C Motif Chemokine Receptor 1)(Lyve1^lo^MHC-II^hi^) and Lyve1^hi^MHC-II^lo^CX3CR1^lo^ (Lyve1^hi^MHC-II^lo^) (42). However, whether peritoneal cavity also contain these parallel resident macrophage subpopulations need to be explored furthermore.

### 2.1 The renewal of peritoneal macrophage subpopulations.

LPMs arise from embryonic progenitors which emerge earlier than hematopoietic stem cells (43). Experiments and studies have confirmed that LPMs are long-lived cells and have the ability of self-renewal locally (35, 44). Early studies suggest that throughout adult life only a minimal part of LPMs are replenished by circulating monocytes in the steady state (34). However, recent studies using tissue-protected bone marrow chimera and fate mapping techniques have shown that Ly6C^+^ monocytes continuously extravasate from blood vessels into the peritoneal cavity through a CCR2-dependent mechanism to replenish CD11c^+^ and CD11c^-^ F4/80^lo^ macrophages during the aging process, with at least a small portion of F4/80^lo^ monocytes acquiring an F4/80^hi^ phenotype (36). This implies that the self-renewal nature of LPMs is not sustained throughout life and that they are progressively displaced by circulating monocytes with age (36).

Unlike for most other tissue resident macrophages, the turnover of F4/80^hi^CD102^+^ (intercellular adhesion molecule 2, ICAM2) peritoneal macrophages from bone marrow-derived macrophages is highly sex dependent, with high or low rates in male or female mice, respectively. Interestingly, the difference in turnover occurs after sexual maturity. In four-week-old Rosa26LSL-eYFP fate-mapping mice, the number of LPMs with eYFP labeling was not significantly different between male and female mice, both approaching 28%. However, by 16 weeks of age, the turnover of LPMs in male mice reached 60%, nearly twice that in female mice, which was 32% (45). What’s more, further studies demonstrated that female and male monocytes have an equal ability to translate into F4/80^hi^ CD102^+^ macrophages in the male peritoneal cavity (45). Thus, it’s the peritoneal local environmental signals that regulate dimorphic effects on peritoneal macrophage replenishment, while the molecular mechanisms underlying the turnover are still unclear.

SPMs and F4/80^int^ intermediate macrophages arising from HSCs, are short-lived cells. Circulating monocytes are responsible for their renewal and peritoneal inflammatory stimulations can make them expand massively (46). Although a small part of F4/80^int^ macrophages get the opportunity to give rise to LPMs, the expression of phagocytic receptor T-cell membrane protein 4(Tim4), a cardinal features of LPMs, is not universally adopted by their descendants (36). In summary, the peritoneal cavity harbors both embryonic-derived and HSC-derived macrophage populations that exhibit distinct renewal dynamics regulated by local microenvironmental cues. Notably, peritoneal macrophage turnover rates demonstrate age- and sex-dependent variations, likely mediated by hormonal influences and cumulative inflammatory exposure.

### 2.2 The phenotypes characteristics of macrophage subpopulations

Macrophage subpopulations present different phenotypes characteristics. LPMs express high levels of F4/80 but low levels of MHC-II, while SPMs express low levels of F4/80 but high levels of MHC-II (47). F4/80^int^ intermediate macrophages gradually acquire the phenotypes characteristics of LPMs and lose the molecular markers of SPMs. Three macrophage populations express CD11b, a cardinal features of myeloid cells.

Furthermore, LPMs express high levels of CD64 (the high affinity IgG receptor) and Mer tyrosine kinase (MerTK), two markers that are now commonly used to identify tissue macrophages, as well as CD102 (intercellular adhesion molecule 2, ICAM2), which has emerged as a signature marker of LPMs (48, 49). Additionally, LPMs also express adhesion and localization molecules, such as CD49f, CD11b, CD73, and CD62P, dead cells recognition and removal molecules, such as CD93, CD36, CD163, macrophage receptor with collagenous structure (MARCO), macrophage scavenger receptor 1 (MSR1), the scavenger receptor Tim4, and MerTK, immune inhibit molecules, such as V‐set immunoglobulin domain‐containing 4 (VSIG4), and other special molecules, such as CD9, CD64, TLR4, TLR7and the M-CSF receptor CFSR1 under the control of cues from the peritoneal microenvironment (18, 32, 47, 50, 51).

On the contrary, most of these markers are not expressed by SPMs at any significant level. SPMs are monocyte-derived cells which highly express the chemokine receptor CCR2. CCR2 plays a critical role in the recruitment of monocytes during peritoneal inflammatory responses and the number of SPMs significantly reduces in the peritoneal exudate of CCR2 KO mice (47). Then, studies have revealed that SPMs are acutely dependent on CSF1 for their development or maintenance (52). And they are heterogeneous in terms of CD11c expression, which was previously considered a dendritic cell-specific marker. Furthermore, the CD11c^+^ fraction is partially affected by Flt3L deficiency and contains both CCR2-dependent and CCR2-independent cells (53). Moreover, a small portion of F4/80^lo^MHCII^+^CSF1^+^ cells express Zbtb46, which has been used extensively to identify cells of the DC lineage (53, 54). Therefore, the F4/80^lo^MHCII^+^CSF1^+^ compartment may contain a small population of DCs which are also monocyte-derived (32). CD226 (DNAM-1), CD206 (mannose receptor) and the immunoregulatory cytokine resistin-like molecule α (RELMα) are useful markers of mature F4/80^lo^MHCII^+^CSF1^+^ macrophages in serous tissue. RELMα expression appears to best discriminate macrophages from the DC component of F4/80^lo^MHCII^+^CSF1^+^ cells (32, 36). As for F4/80^int^ intermediate macrophages, which possess a transitional macrophage phenotype characteristic, expressing a high level of MRC1 and LYVE1, but not expressing GATA6 (43, 55).

Transcription factors are vital for macrophage-specific gene expression, and macrophage subpopulations express distinct transcription factors which regulate the development and differentiation of macrophages. LPMs rely on the zinc finger transcription factor GATA6 for acquiring tissue-special functional features, keeping self- homeostasis and localizing into the peritoneal cavity, while SPMs rely on interferon regulatory factor 4 (IRF4) for maintaining alive and differentiation (56–58). And the expression of GATA6 is negligible in SPMs, thioglycolate-induced peritoneal macrophages, and neutrophils. Retinoic acid, a metabolite of vitamin A produced by mesothelial and fibroblastic stromal cells expressing Wilms’ tumor 1 in the omentum and visceral adipose tissues, is a pivotal driver of GATA6 expression by activating retinoic acid nuclear receptors (47, 59, 60). The production of retinoic acid is controlled by two rate-limiting enzymes, RALDH1 and RALDH2 which is driven by Wilms’ tumor 1 transcription factor. Therefore, by regulating the available of retinoic acid to reverse the transcriptional program of LPMs is feasible (48). LPMs can downregulate the expression of GATA6 in the alveolar space and acquire the alveolar macrophage transcriptional profiling (61). What’s more, the absence of GATA6 results in a higher expression of some proteins, such as CD206 and LYVE, while decreasing the expression of CD73 (47, 62). This is in line with the functional plasticity of macrophage exposed to different environmental signals. Interestingly, global or myeloid-specific deletion of CCAAT/enhancer binding protein (C/EBP) β affects the survival, proliferation and function of F4/80^hi^ LPM macrophages (57). Thus, there are not only one way of regulating the transcriptional program of LPMs.

Taken together, special tissue signal molecules from the peritoneal microenvironment trigger the expression of different transcriptional factors, leading to the initiation of the transcriptional programs. Eventually, macrophage subpopulations present different phenotype characteristics. The signaling pathways between transcription factors and phenotype characters need to be further clarified to assist us in developing new strategies for adhesion protection.

### 2.3 The functional heterogeneity of macrophage subpopulations

Macrophage subpopulations have different functional features. LPMs express a range of receptors that sense physiological parameters, such as molecular signals associated with apoptotic, damaged, or unfit cells. This endows them with the ability to maintain peritoneal homeostasis and provide the first line of defense against life-threatening pathologies of the peritoneal cavity, such as abdominal sepsis, peritoneal metastatic tumor growth, or peritoneal injuries (35, 63). Every day, billions of cells undergo apoptosis at a steady state mammal body. The apoptotic cells (ACs) can release “find-me”, “eat-me” and “digest-me” signals that recruit phagocytes to engulf and digest apoptotic cells (64). As peritoneal resident macrophages, LPMs act as the primary initiators of ACs and express a high level of TIM4, which can bind the phosphatidylserine (PtdSer) released by ACs and promote the engulfment of LPMs (65). Cues from the peritoneal microenvironment can program LPMs for “silent” clearance of ACs to avoid the inflammatory response. This process is regulated by the transcription factors KLF2 and KLF4, which inhibit TLR9 expression and drive the expression of many AC clearance program genes (51). Moreover, LPMs are a physiological main source of CXC-chemokine ligand 13 (CXCL13) within the peritoneal cavity and can recruit B1 cells from the bloodstream into omental lymphoid aggregates, termed “milky spots”, and then move into the peritoneal cavity. B1 cells are peritoneal resident immune cells and occupy the majority of B cells cluster. What’s more, B1 cells express large amounts of surface IgM antibodies, known as natural antibodies, which are present in the serum of unimmunized mice to provide protection from bacterial infections. Some studies have shown that the number of B1 cells is markedly decreased and that the formation of milky spots is impaired in CXCL13-deficient mice. These changes lead to impaired production of natural antibodies and decreased peritoneal immunity (66). Therefore, LPMs play a crucial role in maintaining the population of peritoneal lymphocytes and in the development of the lymphoid network (27, 66, 67).

Compared to LPMs, SPMs have a weaker ability to engulf apoptotic cells, but they can produce much higher levels of nitric oxide and have a greater capacity to phagocytose bacteria. Under conditions of damage, monocytes can be recruited from the blood to tissues and mature into macrophages over a period of 2–3 days (68). At steady state, the number of SPMs is insignificant, and their resting-state functions are rarely studied; therefore, it is speculated that SPMs mainly play a role after inflammation occurs. Moreover, in the various complicated pathological status, the functions of different macrophage subpopulations are more heterogeneous, which are further elaborated in the following.

### 2.4 The heterogeneity of human cavity macrophages

Based on the results of single-cell RNA sequencing (scRNA-seq), the human cavity macrophages are cataloged into eight clusters: MRC1^+^LYVE1^+^CD163^+^ macrophages, GATA6^+^SELP^+^ macrophages, GLUL^+^CD163^+^FABP5^+^ macrophages, MARCO^+^ macrophages, IL1β^+^ macrophages, interferon-stimulated genes (ISG)^+^ macrophages, CCR2^+^ macrophages and CD1C^+^CD14^+^ macrophages (62). MRC1^+^LYVE1^+^CD163^+^GATA6^-^macrophages are the prominent subset in the human peritoneal cavity, matching with mouse CD206^+^ LYVE1^+^ GATA6^-^ cavity macrophages, representing a transitional differentiation stage that occurs before Gata6 expression (49). The proportion of GATA6^+^SELP^+^ macrophages is less than 5% in the human peritoneal cavity, but their counterparts simultaneously expressing GATA6 and SELP in mouse, are the prominent subset, up to approximately 90% (49, 62). Interestingly, the longevity marker, TIMD4, are mainly expressed by CD206^+^ and GATA6^+^ macrophages. Then CD1C^+^CD14^+^ cluster is another prominent macrophage subpopulation enriched in FCGR1A encoding CD64, FCGR3A encoding CD16, FABP5, CSF1R (encoding CD115), CD63 and CD163 and MAFB, which are traditional genes associating with macrophages. GSEA comparison have revealed that the counterpart of CD1C^+^CD14^+^ macrophages is SPMs in mouse and both of them have features of dendritic cells and macrophages (62). As for other human macrophages, including CCR2^+^ or GLUL^+^ macrophages, their counterparts in mouse are still not uncovered. In addition, some other classical macrophage markers have been used to identify human peritoneal macrophages, such as CD11b, CD68, CD86, CD119, Tim4, MerTK, P-selectin glycoprotein ligand (PSGL-1), VSIG4 and CD49F (32, 39, 69).

The body cavity in most multicellular organisms harbors immune cells that clear pathogens and facilitate repairment of cavity injury. In mammals, the first line of immunity in the peritoneal cavity is fundamentally based on phagocytic and antibody-mediated defense mechanisms supported by LPMs and B1 cells. In mouse, LPMs are the prominent components of peritoneal defenses at steady state, and they play a crucial role in sensing and clearing stimuli from inside or outside the body to the peritoneal cavity. Similarly, in human, the peritoneal, pleural, and pericardial cavities are filled with vast numbers of cavity macrophages, and some of them have been found counterparts in mice, while others haven’t. Although these immune cells seemingly perform the same effect of defensing against pathogens and prompting injury impair, the expression pattern and classification of the macrophages are diverse in different species. Up to now, the most of theoretical findings about cavity macrophages comes from the researches referring to the mouse, which include the turnover, the gender differences, and the various functions of cavity macrophages in the homeostasis and pathological states. The clusters of human cavity macrophages are obviously different from the mouse, so it still remains to be further explored whether these studies translate directly to humans. These findings underscore the need to investigate the characteristics and functional adaptations of human peritoneal macrophages across various disease states. Importantly, macrophage subpopulations exhibit distinct transcriptional profiles and specialized functions shaped by their tissue-specific niches. Throughout mammalian lifespan, both LPMs and SPMs play essential yet complementary roles in preserving peritoneal homeostasis (**Table 1**).

**Table 1:** Peritoneal macrophage subsets in different species.

Species	Subsets determined by flow cytometry	Markers	Proportion	Subsets determined by single cell RNA sequencing	References
Sea urchin	large phagocytes	Coelomocytes subsets based on size, structure, and function	71%	none	(152, 153)
	Small phagocytes		2%		
	Red spherule cells (RSCs)		7%		
	Colorless spherule cells (CSCs)		Total 20%		
	Vibratile cells				
mice	Large peritoneal macrophages (LPMs)	Identify makers: F4/80, CD64, MerTK, CD102 CFSR1 Adhesion molecules: CD49f, CD11b, CD73, and CD62P Dead cells recognition molecules: CD93, CD36, CD163, MARCO, MSR1 Scavenger receptor: Tim4, MerTK immune inhibit molecules: VSIG4 other molecules: CD9, CD64, TLR4, TLR7	90%	MHC-II^hi^ macrophages; CCR2^+^ macrophages TLF^+^ macrophages Lyve1^lo^MHC-II^hi^CX3CR1^hi^ macrophages Lyve1^hi^MHC-II^lo^CX3CR1^lo^ macrophages	(31, 32, 36, 39, 41, 42)
	Small peritoneal macrophages (SPMs)	CCR2, MHCII, CSF1, CD226, CD206, RELMα,CD11b, CD11c	10%		
	intermediate macrophages	MRC1, LYVE1, CCR2, CD11b	–		
human	CD14^++^CD16^-^	CD11b, CD11c, CD64, CD116, CD119, HLA-DR	40%	MRC1^+^LYVE1^+^CD163^+^ macrophages, GATA6^+^SELP^+^ macrophages, GLUL^+^CD163^+^FABP5^+^ macrophages, MARCO^+^ macrophages, IL1β^+^ macrophages, interferon-stimulated genes (ISG)^+^ macrophages, CCR2^+^ macrophages CD1C^+^CD14^+^ macrophages.	(62, 69)
	CD14^++^CD16^+^	CD40, CD86	40%		
	CD14^high^CD16^high^	CD80, CD86, CD206	20%		

## 3 Peritoneal macrophages recognize serosal injury

In the steady state, macrophages are free-floating in the peritoneal fluid without attachment to the mesothelium (18, 21). Imaging of macrophages with an intravital microscopy model reveals that the migration rate of macrophages in the peritoneal cavity is very fast, up to 800 μm/s. Additionally, the migration takes place in a random and wholly passive manner that relies on the dynamics flow of peritoneal fluid (18). Therefore, macrophages contribute to constructing the first line of immunity in the peritoneal cavity and are capable of rapidly reacting to any pathogens and serosal injuries. Furthermore, activated macrophages form the aggregates and encompass the pathogens or injuries, or engulf pathogens and die, or move to the omentum and present antigens, and so on, which will result in MDR and initiate and regulate the tissue repair.

### 3.1 The recognition of injury stimuli

Injury stimuli is various, and these proinflammation molecules are different with each other and can be classified as damage-associated molecular patterns (DAMPs), pathogen-associated molecular patterns (PAMPs) and lifestyle-associated molecular patterns (LAMPs) (70). Without pathogens and their products, sterile operations or chemical stimuli can lead to sterile injury of peritoneal cavity. Damaged and necrotic cells, such as mesothelial cells, parenchymal cells and mesenchymal cells, release numerous DAMPs, which are able to activate coagulation responses, serine kinases of kinin, and complement cascades, resulting in the generation of cytokines and inflammatory mediators in the early stage (70–72). DAMPs consist of many types of molecules, categorizable into two types: intracellular DAMPs and extracellular DAMPs. Hence, the receptors of DAMPs are also diversity and consist of pattern recognition receptors (PRRs), such as Toll-like Receptors (TLRs), C-type lectin receptors (CLRs) and cytoplasmic Nod-like receptors (NLRs) and non-PPRs, such as CD44, CD91 and integrins (71–73). Furthermore, many studies have confirmed that the activation of macrophage cells is dependent on various DAMPs, such as the binding of ATP and P2X7 receptors under sterile inflammation conditons (18, 70, 74). PAMPs refer to infectious microbes or their conserved microbial products, such as lipopolysaccharide (LPS). PAMPs activate PRRs, resulting in leukocyte trafficking and inflammation initiation (70, 75). LAMPs refer to an eliciting immunostimulatory molecular pattern, which is neither clearly pathogen nor damage associated, while associate with modern lifestyle (70).

LPMs are the first responders to mesothelium injuries in the peritoneal cavity. Neutrophils emerge in the injured area more than 40 minutes after the injury occurred, slower than LPMs. In the steady peritoneal fluid, LPMs are dominant, while their presence diminishes significantly by 4 hours post-surgery, accounting for merely 1% of the total peritoneal cell population. Furthermore, their disappearance becomes more significant and is even undetectable by 24 hours after the surgical procedure (76). The phenomenon known as MDR, which is initially described fifty years ago, is an inflammatory response to harmful stimuli in the peritoneal cavity (77, 78). Following the injection of the chemical agents or sterile injuries, macrophages are disappearance from peritoneal lavages, while there is a sharp rise in the quantity of inflammatory monocytes, indicating a rejuvenation of immune cells and enhanced effector activity within the peritoneal cavity (47, 77, 79). SPMs are typically difficult to detect in homeostasis conditions while the influx of monocytes leads to a rise in SPMs to comprise 3% of the peritoneal cell population within 24 hours after surgery, with a peak observed on day three (76). Hence, macrophage subpopulations show distinct dynamics features and exert different functions facing challenges.

Both histological investigation and intravital microscopy model reveal that abundant macrophages attach to the injury sites of serosal (18, 76). As follows, it’s possible that the disappeared peritoneal macrophages contribute to the development of cell aggregates on the injury sites. In addition, inflammatory stimuli such as the bacillus Calmette-Guerin vaccine, Escherichia coli (E. coli), LPS, zymosan, thioglycolate or Trypanosoma cruzi have also been confirmed to result in MDR (14, 79, 80). However, the mechanisms underlying MDR exhibit heterogeneity across experimental models. Depleted macrophage populations primarily undergo three distinct fate pathways: (1) formation of multicellular aggregates adherent to mesothelial surfaces, (2) migration to omental milky spots, or (3) pyroptosis-mediated programmed cell death (18, 47, 81). What’s more, many studies have revealed that MDR performs a crucial role in tissue repair and protecting the peritoneal cavity from pathogen infections, sterile injuries, and foreign bodies. Following, we will discuss the dynamic features and the role of activated macrophages under invasion by diverse stimuli (**Figure 1**).

### 3.2 Macrophage aggregates after serosal injury

In a delayed-type hypersensitivity mouse model (78), MDR occurs promptly within 6 h after the injection of tuberculin, and almost all macrophages disappear from the peritoneal lavages by forming aggregates and attaching to the mesothelium (82). Interestingly, LPMs return to the peritoneal fluid 24~48 hours after the initiation of MDR due to the detachment and scattering of the aggregates. Recent studies have replicated this phenomenon in an abdominal sepsis model based on intraperitoneally injection of a nonlethal dose of E. coli (14). Whole-mount immunofluorescence and confocal microscopy are used to visualize the peritoneal wall, revealing that dense macrophage multilayered cellular structures ranging from small to large on the inner face of the peritoneal wall. The formation of this mesothelium bound aggregates is driven by LPMs rather than B1 cells or neutrophils, although these cells make up to 50% of the aggregates, and the absence of B1 cells and neutrophils don’t prevent the formation of macrophage aggregates. Moreover, lymphoid cells, B2 cells, eosinophils, monocyte-derived cells and mast cells are also present in the aggregates (14). In addition, LPMs enriched millimeter-sized aggregates are found in peritoneal lavages 3–5 hours after intraperitoneally injection of zymosan. These free-floating aggregates also contain abundant neutrophils with a obviously low presence of B cells. Of note, In CCR2^-/-^ mice, a well-developed fibrin network still presents in the macrophage aggregates 4 days after E.coli infection, but in WT mice, the fibrin network is clearly degraded (14). Thus, in the late stages of inflammation, monocytes play a critical role in degrading the fibrin network, scattering the aggregates and inflammation resolution. In summary, we suggest that LPMs play a crucial role in monitoring the invasion of stimuli, rapidly reacting to injury stimuli and maintaining the homeostasis of the peritoneal cavity while monocytes possess the ability to promote the dissolution of fibrin clots during the dissipation of inflammation.

Macrophage aggregates play a critical role in entrapping microbes or toxic particles that invade the peritoneal cavity. In the zymosan-induced MDR model, peritoneal macrophages encompass almost all zymosan particles and phagocytose a small part of zymosan particles, but more zymosan particles are not engulfed. In comparison, in the E. coli-induced MDR model, numerous macrophages engulf the bacteria, and no bacteria presents outside of the macrophages (14). Interestingly, these LPMs containing bacteria are mainly located in the central area of the aggregates and surrounded by immune cells not containing bacteria. Blocking the formation of macrophage aggregates with heparin after E. coli injection led to a sharp increase in the number of colonies forming unit accompanied by a dramatic decrease in survival. Similar results are also observed in mice treated with clodronate-loaded liposomes (21, 83). In addition, applying hirudin or knocking out Tln1 alone has a weaker effect on the clearance of bacteria, but the combination of the two interventions has an impact similar to that of treatment with clodronate-loaded liposomes. Thus, the macrophage aggregates are crucial for encompassing zymosan particles and ingesting, digesting, and eliminating bacteria. On the contrary, some studies reveal that Staphylococcus aureus can infect LPMs and serve them as an intracellular reservoir for survival and reproduction, eventually leading to LPMs death (84). Furthermore, LPMs help S.aureus to delay the neutrophilic response and allow its dissemination. While SPMs and neutrophils are the real killers of S. aureus. This suggests that macrophages are not able to defend all kinds of pathogens, and different immune cells are needed for different pathogens.

Although macrophage aggregates play a pivotal role in restricting the dissemination of pathogens, they may also lead to adhesions formation upon peritoneal infection. Two outcomes are associated with the degree of infections and immune responses. If all pathogens can be rapidly killed and effectively controlled, these aggregates will cease to expand or even resolve. But if pathogens aren’t effectively limited, macrophage aggregates will continuously develop to form super-aggregates, which serve as a physical scaffold of peritoneal adhesion. Interestingly, CCR2^+^ monocyte-derived cells are crucial for extracellular fibrinolysis via the integral membrane receptor Plg-RKT, which modulate the activation of plasminogen. In CCR2^-/-^ mice, the disruption of macrophage aggregates is significantly hindered. Furthermore, a study confirmed that CCR2^+^ monocyte-derived cells play a protect role in surgery-induced adhesions (38). Thus, both LPMs and monocyte-derived cells play an important role in adhesion formation. Subsequently, monolayer mesothelial will gradually cover the super-aggregates and then collagen deposition reinforces the adhesion (55). The mechanisms of adhesion in peritoneal sterile injuries is resemble in peritoneal infection which is further reviewed in the following.

### 3.3 Macrophage pyroptosis after serosal injury

Pyroptosis, an innate immune response, is a form of programmed necrosis accompanied by the release of cellular contents and cytokines that results in strong inflammatory responses. Pyroptosis serves as a crucial natural immune defense mechanism that specifically combats infections and addresses endogenous danger signals (85). A nonlethal dose E.coli can result in ~40% LPMs pyroptosis (14, 86, 87). However, the pyroptosis is not due to the ingestion of bacteria, rather, it’s executed by gasdermin D which is cleaved by inflammatory caspases., such as caspase-1, -4, -5 and -11 (88–91). FACS analysis confirms that the number of dead LPMs in the early stage of E. coli infection is significantly restored by proliferation of residual macrophages (14). Moreover, in order to track the fate trajectory of monocytes, a team constructs monocyte fate-mapping models based on the history of Ms4a3 expression. Then they reveal that no monocytes-derived macrophage (moMac) replaces LPMs in the LPS-induced peritonitis model. In contrast, in the thioglycolate-induced or clodronate-induced peritonitis model, LPMs dramatically decreases and then replaced by moMacs (92). Thus, it’s possible that the degree of LPMs decline is the main factor that determines the ability of moMacs to replace LPMs. For instance, low-dose zymosan-induced MDR is limited and transient, while high-dose zymosan causes most LPMs abolition. The former restore the population of LPMs via proliferation of residual LPMs, but the latter needs moMacs to replenish (93). In fact, it’s the availability of tissue-special niches decides whether moMacs develop into LPMs or not (94, 95). Hence, If LPMs are just activated by inflammatory stimuli, they will detach from the mesothelium and return to the peritoneal niches in the end stage of inflammation. If a part of LPMs pyroptosis, residual LPMs will show strong proliferation ability. In the end, only most LPMs death, moMacs can get chances to enter the niches and develop into LPMs.

### 3.4 Migration to the omentum

The disappeared macrophages also migrate into omental milky spots via discontinuities in the mesothelium (14, 27, 80). LPMs with peritoneal antigens that migrate into the omentum can induce a further immune response (27). Recent studies have confirmed that the migration of LPMs to the omentum is indispensable for the recruitment of B cells, T cells, monocytes and neutrophils to omental milky spots after E. coli infection. Furthermore, depleting macrophages with clodronate impaired the development of milky spots, as evidenced by the smaller size of these spots and the decreased weight of the omentum compared to those of the control mice (14). Hence, macrophages migrating into milky spots is an important immune response for the defense against pathogens invasion. According to reports, many factors such as zymosan, LPS and E. coli can trigger this kind of migration. Interestingly, more macrophages engage in the macrophage aggregates, while less macrophages migrate to the milky spots in the zymosan mouse model. In addition, blocking Tln1 obviously inhibits this reaction (21, 80). Likewise, anticoagulation drugs, such as heparin and hirudin can also alleviating this response in the mouse model of LPS or E. coli intraperitoneally injection (47). However, compared with hirudin, heparin presents a more significant suppress effect (96). Some studies have argued that low-dose zymosan is not able to induce macrophages migration to the omentum. It’s possible that low level of inflammation maybe insufficient to induce ample LPMs leave niches to omentum to recruit more immune cells.

To summarize, LPMs are the first sensors in peritoneal injuries. They recognize the serosal injury and respond rapidly, and MDR is the classic response pattern which initiates elimination of pathogens and promotes tissue repair. However, MDR is a very complicated process involving interactions among the coagulation pathway, the migration of LPMs, the replenishment of the LPM pool and the immune response. It plays an essential role in protecting the peritoneal cavity from inflammatory stimuli by promoting the formation of mesothelium-bound, fibrin-dependent aggregates and the development of milky spots. Reactions similar to those for MDR bacteria may also occur in other cavities. The mechanisms underlying MDR need to be explored further

**Figure 2:**
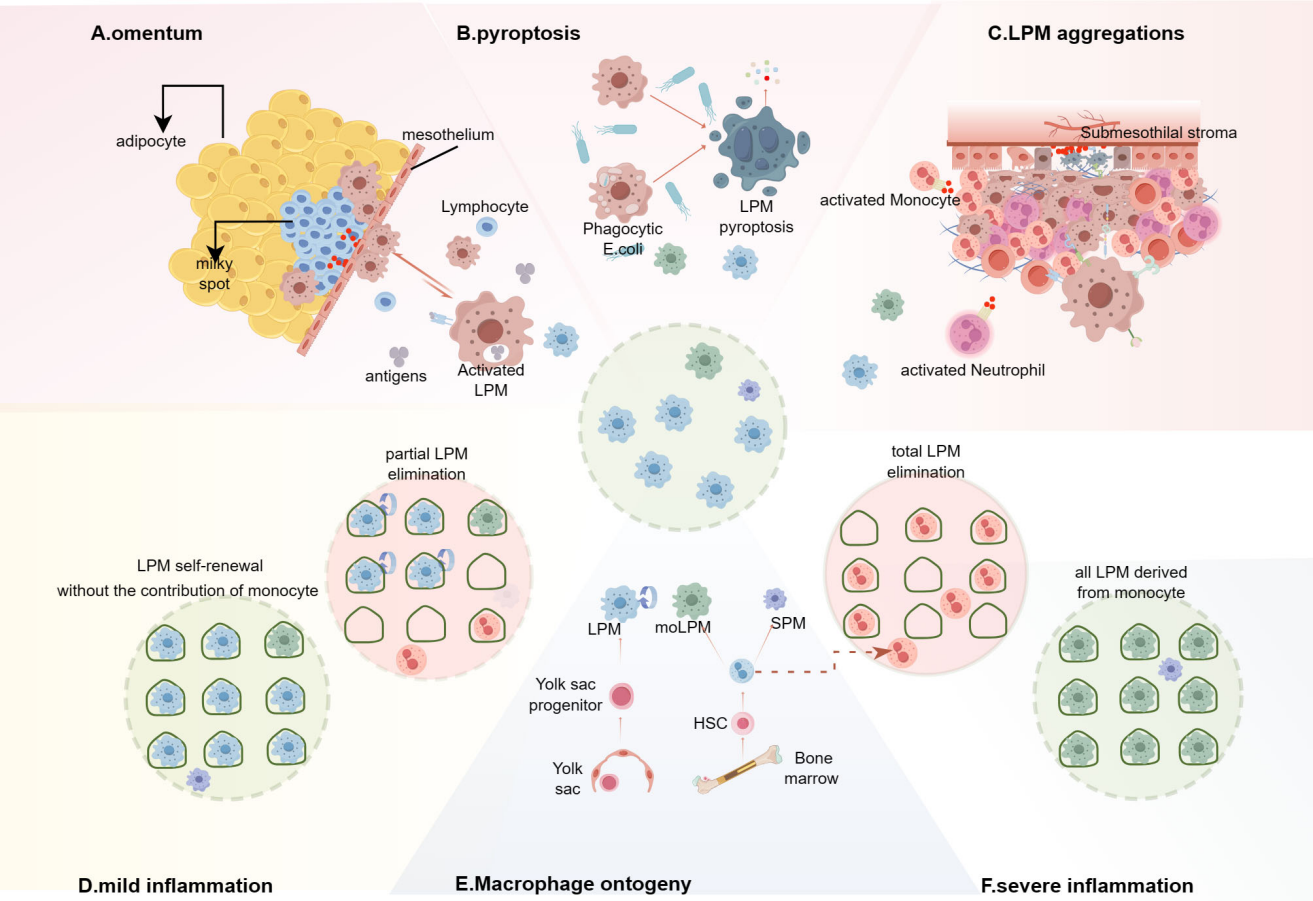
Integrated model of macrophage disappearance reaction (MDR) and the replacement of LPMs in inflammation. **(A–C)** In different MDR models, the mechanisms are not consistent and the disappearing LPMs mainly migrate into the omental milky spots, or pyroptosis, or form multicellular aggregates bound to the mesothelium lining the peritoneal wall and the peritoneal organs. **(A)** Macrophages that capture or engulf peritoneal antigens migrate into the omental milky spots and secret chemokine, such as CXCL13, to recruit T cells, monocytes and other immune cells. **(B)** *E.* coli infection induces LPMs pyrotosis regardless of the ingestion of bacteria. **(C)** The multilayered cellular structure is composed of LPMs, B1 cells, neutrophils, eosinophils, monocyte-derived cells (moCs) etc. and shields the injury mesothelium. **(D, F)** Inflammation response leads to LPMs death, creating niche availability, which is related to the severity of inflammation. **(D)** In mild inflammation without significantly affecting the niche, the remaining LPMs occupy the available niche via proliferation. **(F)** Severe inflammation leads to the disappearance of LPMs, the niche will be eventually filled with monocyte-derived LPMs (moLPMs). **(E)** In the peritoneal cavity of adult mice, the LPMs is made up of cells from two different origins, embryonic LPMs and moLPMs. Embryonic LPMs are progressively displaced by moLPMs with age. (By Figdraw.).

In summary, LPMs serve as primary sentinels for peritoneal injury, rapidly detecting serosal damage and initiating responses. The MDR represents a classic defensive pattern that facilitates pathogen clearance and tissue repair initiation. This complex process involves coordinated interactions between multiple systems: (1) coagulation cascades, (2) LPM migration dynamics, (3) LPM pool replenishment mechanisms, and (4) innate immune activation. Through fibrin-dependent aggregates formation at mesothelial surfaces and milky spot development, MDR provides crucial peritoneal protection against inflammatory insults. Notably, analogous reactions may occur in other serosal cavities beyond the peritoneum. Further investigation is required to fully elucidate the molecular mechanisms governing MDR (**Figure 2**).

## 4 Macrophage aggregates regulate serosal repair

The coelomic cavity of sea urchins harbors abundant immune cells, called coelomocytes (97). These cells have the functions of both platelets and phagocytes so that they are indispensable for the clearance of pathogens and wound healing. By forming aggregates, they provide a physical barrier to avoid potential leakage after injury to the coelomic cavity (98–100). In mammals, LPMs play a pivotal role in establishing the first line of immunity within the peritoneal cavity, similar to the primitive defense mechanisms maintained by various populations of coelomocytes present in the coelomic cavity of invertebrates. Likewise, LPMs have the functions of phagocyte and extravascular platelet. Therefore, LPMs are crucial for maintaining the integrity of the peritoneal cavity, eliminating pathogens and promoting tissue repair.

The forming of macrophage aggregates on the serosal injury site is important for the injury repair, affecting whether the injury is completely healed and the extent of peritoneal adhesion formation. The process is quick and involves in many engagements. To begin with, injured tissue release DAMPs, such as ATP, which activates free LPMs. Then, LPMs attach to the wound bed and undergo a morphological change within few seconds. Subsequently, more LPMs attach to these already-attached macrophages via class A scavenger receptors, such as MSR1 and MARCO. Meanwhile, other recruited immune cells also contribute to the formation of multicellular structure. Then, monolayer mesothelial will gradually cover the super-aggregates and abundant fibrins deposition reinforces the structure. Eventually, there are two different repair outcomes: complete restoration or fibrotic scar formation (18). Studies reveal that two outcomes are mainly associated with the range and degree of injury. In summary, LPMs mainly provide an emergency repair that physically seals potential leaks by forming aggregates reminiscent of platelet aggregation and the number of attached cells is closely relative to sterile injury size.

### 4.1 Focal mesothelium injury

Macrophages can seal the microlesions to prevent the initiation of inflammation. Studies has revealed that Prior activated inflammatory neutrophils can release abundant secondary mediators, such as LTB4, resulting in the swarming behavior of neutrophils, which not only increases the extent of injury, leading to severe local collateral tissue injury but also degrades collagen fibers, leading to the breakdown of coagulated tissue (101–103). Previous studies have confirmed that resident tissue macrophages can cloak microlesions to avoid terminal inflammatory activation of endogenous neutrophils or the presence of neutrophil swarming (101). In addition, the macrophages involved in the formation of cloaking undergo a morphological shift and protrude multiple pseudopods. Therefore, the mechanisms underlying cloaking involve not only the physical protection of damaged tissue but also the process of phagocytosis to eliminate DAMPs (101). Indeed, cloaking plays a crucial role in promoting microlesion repair in a noninflammatory manner.

A focal thermal injury on the mice abdominal wall is indued by using a multiphoton. Upon injury, a few macrophages rapidly attach to the wound area within seconds. Of note, these attached macrophages undergo a morphological change from a round appearance to an elongated epithelioid phenotype. This morphological shift enhances the adhesion ability of macrophages to protect them from detaching under high fluid shear stress, similar to an anchor, and increases the area covered by macrophages to protect the wound, similar to the cloaking. Then, more macrophages directly tether to these already-attached macrophages to form tight aggregates, similar to platelet aggregation and thrombus formation. However, the development of cell aggregates is not infinite and stabilized by 15 minutes after injury. It’s possible that cell aggregates perfectly seal the injury site so the inflammation response is not initiated, or there is a regulating mechanism to control the growth of cell aggregates. Up to now, the mechanisms underlying the development ending of cell aggregates are still unclear. Experiments have revealed that cell aggregates are completely composed of LPMs in the early stage, despite the peritoneal cavity harbors diverse immune cells. Moreover, Inhibition of platelets or neutrophils also had no significant effect on the recruitment of LPMs. Additionally, the recruitment of LPMs is totally passive and depends on the fluid shear flow.

There is no evident chemotactic response detected at any stage of the recruitment procedure. In addition, LPMs express a variety of canonical adhesion molecules, such as integrins (CD29 and CD18), selectins (P- and L-selectin), and immunoglobulin-like adhesion molecules (Icam2 and CRIg), but blocking these molecules does not impair the formation of macrophage aggregates (18, 104). Anticoagulation drugs, such as hirudin and argatroban, only partly alleviate LPMs aggregation, while heparin, a large polyanion, has an obvious effect. On the contrary, by means of polyinosine acid [poly(I)] to block MSR1 and MARCO which are capable of binding to various polyanionic ligands, the size of macrophage aggregates is significantly decreased (18, 105). Notably, poly(I) specifically influences the second tether of LPMs binding to macrophages that are already adhered to the injured serosal tissue, without disrupting the initial recognition or adhesion to the site of injury (18). *In vitro*, experiments confirmed that ATP and Ca^2+^ are able to accelerate LPM aggregation because ATP promotes the migration of MSR1 and MARCO from the intracellular fluid to the membrane and Ca^2+^ are necessary for coagulation response by activating coagulation factors. Suppressing the aggregation of LPMs with poly (I) shows a prolonged healing of the focal thermal injury by 3 days after injury, which should be complete restoration in normal situation, suggesting that macrophage aggregates are necessary for tissue repair.

### 4.2 Restricted mesothelium injury

The size of macrophage aggregates attached to the injury site is closely relative to the extent of damage. In order to image the peritoneal macrophages, a team implant a chronic imaging window on the mouse peritoneal wall. Meanwhile, this operation also creates a severe injury, leading to the activation and aggregation of LPMs in distinct places, such as the window surface, mesothelium or just free-floating in the peritoneal fluid. Then, these cell aggregates merges with each other to form super-aggregates. Of note, the development of cell aggregates induced by severe injury is uncontrollable. Subsequently, super-aggregates continuously merge to form bridges, linking the imaging window and visceral organs, such as the intestine, omentum, or liver, and these bridges will be gradually covered with mesothelium in 3 days (18). Simultaneously, kinds of stromal cell such as fibroblasts, stem cells, infiltrate into bridges and secret excessive ECM components after 7 days, marking adhesion formation. The peritoneal button model is a kind of stable peritoneal adhesion formation model. In this model, the size of macrophage aggregates significantly reduce via blocking scavenger receptors with poly(I), and the number and range of adhesions also significantly decrease within 7 days after injury (18, 106). Furthermore, using clodronate liposomes to completely ablate peritoneal macrophages shows a more significant effect in alleviating PAs (18). What’s more, depleting neutrophils has no effect on the formation of PAs. Due to the time point of estimating PA is 7 days after operation, no significant extension of healing is observed.

Collectively, compared to focal thermal injury, restricted mesothelium injures, such as a laparotomy or foreign body implantation, results in excessive LPMs attached to the wound bed and then forming super-aggregate which gradually develops into PAs. Inhibition the formation of super-aggregates via blocking the MSR1 and MARCO, or depleting LPMs, gets a significant effect on alleviating the degree of PAs. We maintain that an iatrogenic procedure results in a broader spectrum of injuries compared to focal injuries, and that LPMs are insufficiently to cover all of these injuries promptly, leading to LPMs overreaction. Meanwhile, there is an absence of effective regulatory mechanism to prevent the undue accumulation of LPMs. As follows, targeting macrophage is potential therapies for preventing PAs.

### 4.3 Extensive mesothelium injury

Extensive mesothelium injury leads to adhesion formation. Under the steady state, there are about 1.73×10^6^ LPMs in the peritoneal fluid of mice (21). Therefore, if the extent of the injury is excessive, there will be not enough number of LPMs to provide adequate coverage for injury area. Some studies have confirmed that the development of PAs results from the inadequacy macrophage barrier to sufficiently cover all exposed fibrin clots, though the injury area is only covered by a monolayer of LPMs (76). Moreover, Immunofluorescence images show that few LPMs present at the site of adhesion formation while no adhesion forms in areas adequately covered by macrophages. Enhancing macrophage barriers with IL-4 was able to alleviate adhesion, but depleting LPMs with clodronate liposomes or inhibiting the binding between LPMs and fibrin clots with a CD11b blocking antibody significantly promoted the formation of peritoneal adhesion (76). In addition, they reveal that monocytes-derived macrophages are not capable of forming an anti-PAs cell barrier (76) In summary, the number of LPMs is limited, so if the area of injury is too large, the number of LPMs will be not enough to perfectly cover the injury. In this situation, increasing the number of LPMs is an effective way to avoid the formation of adhesions.

In conclusion, the migration of LPMs and the formation of macrophage aggregates appear to be indispensable steps in the process of peritoneal tissue repair. The roles of LPMs in sterile peritoneal injury repair are diverse and depend on the severity of the injury. They can promote the formation of adhesions in severe injury and can also protect focal damaged tissue from further injury by shielding exposed fibrin clots to inhibit neutrophil swarming. It is possible that mammals have evolved a mechanism for repairing focal injuries, but severe injuries, such as abdominal surgery, are beyond the repair ability of this mechanism and eventually lead to scar repair.

### 4.4 Cavity parenchymal organs injury

The behaviors of LPMs are not consistent among the different injury sites. Using a heated thermal probe to induce liver capsular injury, the intact mesothelium was destroyed, and intravital microscopy model imaging over long periods showed that Kupffer cells did not move into the injury sites through the vasculature (107). In contrast, LPMs directly migrated to the injury sites from the peritoneal cavity and adhered to the apex of necrotic cells within 1 hour after injury (83). However, immunofluorescence staining reveals that LPMs don’t appear inside the injury site. Interestingly, in the CCl_4_-induced acute hepatotoxicity model, a large number of LPMs invade the liver, and some even reach a depth of 500 μm. LPMs are localized to sterile injury sites in the liver and rely on various DAMPs, including ATP released by necrotic cells and the P2X7 receptor for activating LPMs; subsequently, CD44 binds to the hyaluronan present in the injured areas. Pretreatment with apyrase, an ATP receptor antagonist, hyaluronidase, or an anti-CD44 antibody significantly decrease the number of LPMs recruited to the injury site. Blocking integrins (CD29 and CD18) has no effect on the recruitment of LPMs to liver injury sites (32, 108, 109), which is consistent with the recruitment of LPMs to peritoneal wall injury sites. Interestingly, the proliferation of activated LPMs at the injured site increase, and these activated cells rapidly upregulate the expression of markers of alternatively activated/repaired phenotypes, such as CD273, CD206, and arginase 1, suggesting that LPMs play a crucial role in promoting injury repair (56, 83, 110). Intravital microscopy model revealed that LPMs are able to engulf and disassemble necrotic DNA and promote revascularization in the area of injury. Depleting LPMs with clodronate-loaded liposomes significantly delays tissue repair. The same phenomenon is also observed in Gata6-deficient mice (83). Using a heated thermal probe to induce a focal necrotic lesion on the colon of CX3CR1^GFP/+^CCR2^RFP/+^ mice, intravital microscopy model shows that abundant LPMs aggregated in the center of the necrotic lesion, surrounded by CCR2^+^ monocytes. Pretreatment with clodronate-loaded liposomes increased the clearance of necrotic cells and intestinal tissue repair. In a dextran sodium sulfate induced colitis model, the depletion of LPMs resulted in more severe colitis activity and weight loss (74). Some have argued that treatment with clodronate-loaded liposomes also depletes monocyte-derived macrophages, so the contribution of these cells to tissue repair cannot be excluded (63, 111).

**Figure 3:**
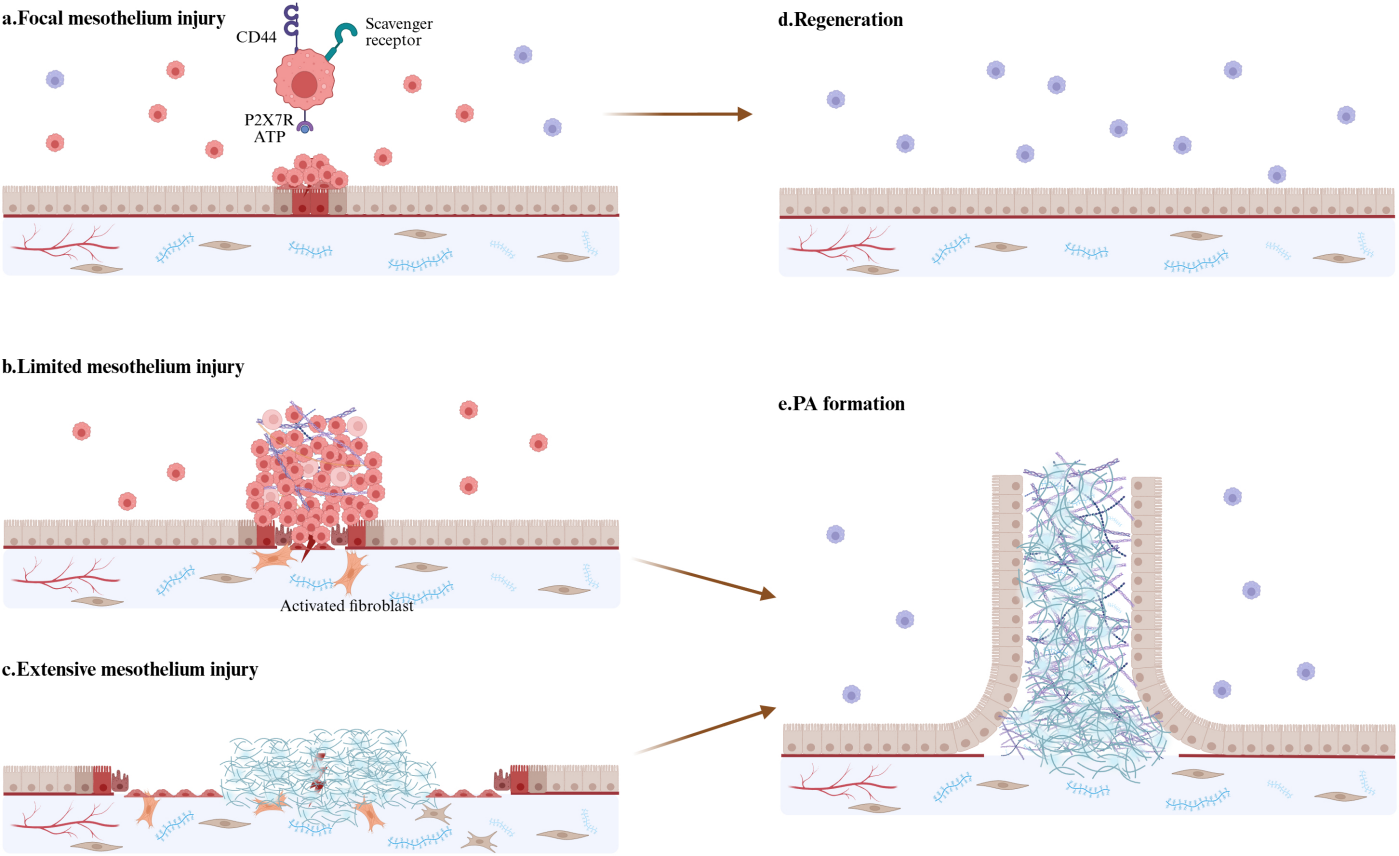
Integrated model of LPM and mesothelium injury. **(a)** LPMs aggregate at sites of mesothelial injury to maintain mesothelial integrity within few minutes. ATP activate LPMs through P2X7 receptors. Class A scavenger receptors-MSR1 and MARCO, mediate LPMs attach to those already-attached macrophages. **(b)** In response to more severe but limited mesothelium injury, LPMs form super-aggregates which often develops into peritoneal adhesion. **(c)** If the area of injury mesothelium is too large, LMPs will be not insufficient to cover the damage and exposed fibrin clots will result in persistent inflammatory response, fibrin deposition, and fibroblast activation—culminating in excessive ECM accumulation and PAs formation. **(d)** Focal mesothelium injury is repaired through regeneration. **(e)** Limited and extensive mesothelium injury lead to uncontrolled inflammation response and ECM deposition. Activated mesothelial cells show strong proliferation and migration abilities and eventually finish the mesothelialization of the adhesion tissue. (By Biorender.).

However, a recent report has cast doubt on the opinion that LPMs migrated into the damaged tissue and played a crucial role in tissue repair (112). They have developed a genetic fate-mapping system, which is used for the construction of the G6Mø-CreER mouse to allow for permanent, efficient and specific lineage tracing of endogenous LPMs. Treating mice with CCl_4_ or acetaminophen to induce liver injury reveals that liver parenchymal tissue present a more significant fibrotic response in the G6Mø-CreER; R26-tdTomato group than in the sham group, but no obvious accumulation of LPMs is observed (83, 112). In the liver cryoinjury model of G6Mø-CreER;R26-tdTomato mice, severe fibrin deposition is observed at the injury sites. In contrast, in the heated thermos probe-induced liver injury model in G6Mø-CreER; R26-tdTomato mice, thicker layers of the mesothelium cover the injured liver regions. Thus, LPMs do not invade deep into the liver for its repair. Although many LPMs aggregate at the injury site, they are mainly on the surface of the thickened mesothelium, and few of them invades deep into the liver parenchymal tissue (112). Additionally, resident macrophages in the pleural cavity minimally invade the lung to repair injured lung tissue. Current experimental evidence demonstrates that neither clodronate liposome-mediated depletion of LPMs nor genetic ablation of GATA6 significantly impairs visceral organ repair processes. These findings suggest the need for further investigation to definitively establish whether LPMs contribute substantially to peritoneal tissue regeneration (**Figure 3**).

## 5 Peritoneal macrophages coordinate the coagulation, inflammation, fibrosis and fibrinolysis, and MMT in PA formation

The formation of PAs involves a complex interplay of inflammatory cascades, coagulation activation, fibrotic deposition, and impaired fibrinolysis. Accumulating evidence demonstrates that distinct subsets of peritoneal macrophages critically orchestrate both tissue repair processes and pathological adhesion development through multiple regulatory mechanisms. As primary sentinel cells, LPMs rapidly detect tissue injury and initiate protective responses. Through controlled aggregation, LPMs form temporary physical barriers that limit excessive inflammation; however, persistent aggregates may instead facilitate adhesion development. Notably, peritoneal macrophages constitutively secrete coagulation factors (e.g., factor V, VII) during homeostasis, maintaining a poised state for rapid hemostatic activation and emergency tissue repair. Macrophages demonstrate potent phagocytic capacity, enabling efficient clearance of pathogens and necrotic debris during acute inflammation while simultaneously releasing immunomodulatory cytokines to coordinate host defense. Notably, their ability to engulf apoptotic immune cells (e.g., neutrophils) through efferocytosis plays a critical role in promoting inflammation resolution and tissue repair. Macrophage subsets exhibit functional plasticity in fibrosis regulation, with distinct populations demonstrating either anti-fibrotic or pro-fibrotic activities. Particularly, macrophage-derived TGF-β serves as a pivotal mediator that both activates collagen-producing fibroblasts and induces MMT, ultimately driving ECM deposition. In the following discussion, we will systematically examine the dual roles of macrophages in postoperative adhesion pathogenesis, focusing on their regulation of fibrotic processes.

### 5.1 The regulation of coagulation response by peritoneal macrophages

Emerging research has established peritoneal macrophages as the important cellular source of coagulation factors within the peritoneal cavity. Gene array analysis shows that LPMs constitutively express high levels of coagulation factor V (FV; F5) in the steady state (50, 108, 113). Using F5^-/-^; AlbF5Tg mice - a genetic model where FV expression is restricted to hepatocytes (via albumin promoter) while absent in all extrahepatic tissues including LPMs – a team observed a 75% reduction in peritoneal fluid FV activity compared to wild-type controls. Besides, LPMs also express the coagulation factors FVII (F7) and FX (F10) (21, 23, 114). This contrasts with systemic circulation, where hepatocytes serve as the primary producers of clotting factors that are largely excluded from the peritoneal space by the mesothelial barrier’s selective permeability. In fact, the coagulation pathway is one of the most highly enriched pathways in LPMs (49). Interestingly, resting LPMs don’t express the F3 gene, but activated LPMs rapidly upregulated it (21). What’s more, LPS stimuli, but not E.coli infection, could also upregulate the expression of the TF gene in LPMs (14, 115).

Early studies demonstrate that the coagulation pathway plays a crucial role in the process of MDR, and the administration of the anticoagulant drugs, such as heparin, hirudin or warfarin suppress MDR in different extensions (78, 116). The dynamics flow of peritoneal macrophages is controlled by the coagulation responses, and fibrinogen staining of LPM aggregates shows that a fibrin network is present between cells. Of note, heparin has both anticoagulation and antiadhesion effects, while hirudin is a very selective thrombin inhibitor that can inhibit the production of fibrin but has no effect on the functions of LPMs (14). However, heparin and hirudin have the same effect on suppressing MDR caused by E.coli injection. And the formation of mesothelium-bound macrophage aggregates is fibrin dependent (14). Besides, the number of LPMs retrieved from peritoneal lavages is comparable in mice treated with hirudin or heparin at 4 hours after E.coli infection (14). However, in the zymosan-induced MDR model, MDR is completely suppressed by heparin but only partially suppressed by hirudin (21). This difference may be caused by the different mechanisms of MDR due to the difference between pathogens and sterile stimuli.

Loss of the integrin activation adaptor talin-1 (Tln1) expression in LPMs of Lyz2^Cre^Tln1^fl/fl^ mice partially suppresses MDR after zymosan injection, and the effect similar to hirudin. But the combination of knockout Tln1 and hirudin can fully reverse MDR, just like heparin (21). Tnl1 is involved in the adherence of LPMs rather than the coagulation process. Therefore, the formation of free-floating macrophage aggregates depends on both fibrin and coagulation. In addition, tissue factor (TF; FIII; F3) initiates the extrinsic coagulation cascade. And MDR is partially suppressed after zymosan injection in Lyz2^Cre^F3^fl/fl^ mice (21, 117, 118). Moreover, zymosan-induced MDR in F5^-/–^AlbF5Tg mice is also partially suppressed to a similar level to that in WT mice treated with hirudin (21). Collectively, these findings establish LPMs as critical regulators of local coagulation cascades within the peritoneal cavity, and coagulation activation plays an indispensable role in MDR, contributing to both pathogen containment and tissue repair initiation.

### 5.2 The regulation of inflammation by peritoneal macrophages

Based on the way of activation, macrophages are traditionally divided into M1/M2 macrophages. M2 macrophages can be further classified into four subsets: M2a, M2b, M2c, and M2d (119, 120). M1-like macrophages are classically activated, pro-inflammatory macrophages with the expression of high levels of CD80, CD86, iNOS (121). M2-like macrophages are alternatively activated, anti-inflammatory macrophages with the expression of high levels of CD163, CD206, Arg1, FIZZ1, and YM1. Of note, M2b macrophages possess pro-inflammatory properties at the same time (122). The polarization of macrophages in the injured area is an important factor affecting the formation of peritoneal adhesion. Previous studies revealed that the counteraction inflammation by diverting M1-like macrophages towards M2-like attenuates postoperative adhesion formation. The polarization of macrophages toward an M2-like phenotype has emerged as a key therapeutic strategy in preventing postoperative adhesions, with multiple novel treatments demonstrating efficacy through this mechanism.

Peritoneal macrophages contain diverse subgroups with distinct expression profiles, origins and functions. The inflammation is mainly driven by circulating monocyte-derived macrophages which are capable of producing high levels of proinflammatory cytokines and chemokines.^2^ In response to PAMPs and DAMPs, peritoneal macrophages quickly initiate inflammatory responses. Activated macrophages secret ample chemokines and cytokines, such as TNF-α, IL-6, IL-12, CCL5, CXCL9, CXCL10 and IL-1β, which are effective to recruit and activate monocytes and leukocytes, such as T cells and B cells to the site of injury. They also perform the function of antigen presentation to help activate adaptive immunity (122, 123).

On the contrary, embryonically derived macrophages prominently play a vital role in attenuating inflammation by producing anti-inflammatory factors such as IL-10 and TGF-β, phagocytosing dead cells, inhibiting leukocyte recruitment through the secretion of matrix metalloproteinases, and promoting tissue repair by producing growth factors and remodeling ECM (123, 124). In addition, macrophages can regulate the function and abundance of specific T cell subsets to attenuate inflammatory responses (125). Of note, the lungs contain a CD169^+^ interstitial macrophage subset which is a major source of IL-10 when infect influenza A virus or bacterial (126).

Macrophages are central players and active participants in all stages of the inflammatory process. However, the molecular mechanisms underlying their ‘switch’ from proinflammatory to anti-inflammatory phenotypes in different conditions are still obscure. Targeting the key molecules of regulating macrophages phenotypes may be potential therapeutics.

### 5.3 The regulation of fibrosis and fibrinolysis by peritoneal macrophages

The key characteristic of fibrosis is that fibroblasts secret abundant ECM components such as fibronectin, collage and laminins. In fact, fibrosis is an important and normal phase of tissue repair. However, the endings of fibrosis are diversity and closely relative with the extension of tissue injury. In general, minor injury results in the transient accumulation of ECM, while severe injury results in the continuous accumulation of ECM. As follows, minor injury acquires the perfect restoration and severe injury leads to the formation of scarring tissue which influences the normal architecture and function of organs (127). Studies shows that tissue resident macrophages are one of the key regulators of fibrosis and involved in multiple phases of tissue repair.

Recent studies confirm that diverse macrophage subsets with different transcriptional program play an anti-fibrotic or profibrotic role in tissue repair (104, 128). Across distinct organs, such as lung, heart, fat and dermis, there are two conserved and independent macrophage subsets, called interstitial macrophage populations, exhibiting distinct the transcriptional program and phenotypes: Lyve1^lo^MHC-II^hi^CX3CR1^hi^ (Lyve1^lo^MHC-II^hi^) and Lyve1^hi^MHC-II^lo^CX3CR1^lo^ (Lyve1^hi^MHC-II^lo^) (42). Both two macrophage subsets arise from Ly6C^hi^ monocytes while they locate in the distinct tissue-special niches. Lyve1^lo^MHC-II^hi^ subpopulations adjacent to nerve bundles and endings possess superior antigen-presentation capacities, but Lyve1^hi^MHC-II^lo^ subpopulations adjacent to blood vessels mainly contribute to the wounding and tissue repair (42). Then, using the mouse model of inducible macrophage depletion to acutely deplete Lyve1^hi^MHC-II^lo^ macrophage subset, the team found that the lung and heart fibrosis is obviously exacerbated in the mouse model of bleomycin-induced fibrosis. Therefore, the Lyve1^hi^MHC-II^lo^ tissue resident macrophages play a vital role in anti-fibrosis and tissue repair. Meanwhile, some studies have confirmed that embryonically derived cavity macrophages are key regulators of fibrosis. Gata6^+^ cavity macrophages in the peritoneal, pleural and pericardial cavity fluid possess the same embryological origin and share a similar transcriptional profile. Pervious work revealed that LPMs directly migrate to the injury liver and promote tissue repair by forming a shield on the injury sites (83). Recent studies demonstrated that in the mouse model of myocardial infarction, Gata6^+^ pericardial cavity macrophages also quickly invade the epicardium where they lost Gata6 expression. What’s more, depleting this macrophage subpopulation results in more severe heart fibrosis (104). Therefore, Gata6^+^ cavity macrophages possess anti-fibrotic properties and cardioprotective functions. On the contrary, by using a novel computational framework, a team revealed a disease-associated, monocyte-derived macrophage subgroup: CX3CR1^+^SiglecF^+^ transitional macrophages. They localized to the fibrotic niche, possessed a transitional gene expression profile intermediate between monocyte-derived and alveolar macrophages and played a role in promoting fibrosis (128). Overall, macrophages with functional plasticity exert different functions in homeostasis, immune regulation, and fibrosis. Further studies need to be done to clarify the spatial-temporal characteristics of peritoneal macrophages to reveal their core functions after serosal injury.

### 5.4 The regulation of MMT by peritoneal macrophages

One report suggests that the initiation of peritoneal adhesion relies on the formation of abundant membrane bridges between mesothelial surfaces (129). Mesothelial cells undergo morphological transformation via Ca^2+^ release and actin remodeling under the stress of injury stimuli. These cells produce many cytoskeletal protrusions, which are able to fuse with each other and transmit cytosolic contents to healthy mesothelial cells, prompting them to acquire the pathological phenotype of adhesion (129). Blocking membrane protrusions with small-molecule inhibitors and blocking antibodies inhibits the formation of postoperative adhesions. We think that mesothelial cells undergo morphological transformation to promote the re-mesothelialization of damaged tissues, providing a physical shield for exposed fibrin clots, similar to LPMs. Furthermore, studies have confirmed that monocytes-derived macrophages can activate mesothelial cells via the CX3CL1–CX3CR1 interaction, leading to the acceleration of peritoneal fibrosis (130). Another study confirmed that monocytes-derived macrophages can produce EGFR ligands, such as Hbegf and Areg, to activate EGFR on the surface of mesothelial cells, leading to collagen deposition and the MMT (23). Thus, it is possible that LPMs play an important role in reducing the morphological transformation of mesothelial cells to the injury site by shielding fibrin clots to alleviate the inflammatory responses and the recruitment of monocytes.

Fibroblasts are the main source of ECM and a range of interactions between profibrotic macrophages and fibroblasts regulate the formation of scaring tissue (131). A recent study revealed that injury tissue releases ATP and LPS, which lead to the expression of amphiregulin by alveolar macrophages. Then, amphiregulin activates the integrin-a_V_ complexes on pericytes and subsequently the release of bioactive TGF-b which induces pericyte into myofibroblast differentiation (132). In addition, profibrotic macrophages are the predominant source of the mitogen Pdgf-aa. Using the antibody blockade of Pdgf-aa significantly inhibits the proliferation of 3T3 fibroblasts in the conditional media of CX3CR1^+^SiglecF^+^ transitional macrophages (128). On the other hand, fibroblasts can generate collagen deformation fields which are critical for the initiation and migration of macrophages in fibrillar collagen matrix (133). Overall, cross-talks between distinct macrophages subsets and fibroblasts are vital for the regulations of tissue remodeling and fibrosis (**Graphical Abstract**).

Taken together, peritoneal macrophages are crucial in every stage of PA formation relying on their various functions associated with coagulation, inflammation, fibrosis and fibrinolysis, and MMT, etc. We suppose that peritoneal macrophages are the core regulatory center, which regulates the immune system to eliminate toxic particles and promote tissue repair, but severe tissue injury leads to their dysfunction. Thus, by controlling the functions of peritoneal macrophages to restore the balance of coagulation, inflammation, fibrosis and fibrinolysis, and MMT is a potential approach for PA prevention.

## 6 Targeting peritoneal macrophages prevents the postoperative adhesion formation

The ideal pattern of anti-PA therapy is to prevent the formation of adhesions without interrupting wound healing. Mechanically, the interactions among coagulation responses, inflammation, and fibrinolysis are prominent factors for the formation of PAs. And on this basis, different pharmacological strategies have been assessed for their anti-adhesive properties. Inert polymers, as the physical barriers, also can be used to prevent or lessen the severity of post-operative adhesions. Now, functional biomaterials, which are designed to act as inert barrier loaded by the effective drug, have attracted great attention in preventing PA. In previous articles, anti-adhesion strategies have been reviewed comprehensively and systematically. In view of the vital roles of peritoneal macrophages in peritoneal injury and adhesion formation, the effect mechanism of many anti-PA strategies is related with peritoneal macrophages, and we summarize the studies on targeting peritoneal macrophages to prevent adhesions.

### 6.1 Drugs targeting peritoneal macrophages

In published literatures, the anti-PA drugs mainly acted on coagulation, inflammation, fibrinolysis, and related cytokines. Parts of these drugs exert the function of adhesion inhibition via targeting peritoneal macrophages.

Heparin, a widely applied anti-coagulation drug, shows a similar anti-adhesion effect to anti-PA membrane Seprafilm without affecting wound healing (2). Some studies have confirmed that heparin could obviously inhibit the formation of macrophage aggregates at the injury sites and alleviate the MDR (18, 21). However, high dose heparin has the risk of continuous bleeding in the wound. Therefore, the primary difficulty in using these anticoagulant medications for anti-PA lies in finding the right balance between anticoagulation and hemostasis.

The combination of AMD3100 (plerixafor) and low-dose FK506 (tacrolimus) can significantly reduce the burden of peritoneal adhesion, while single drug has no effect on avoiding adhesion formation. Mechanically, the combination treatment dramatically promotes macrophages M2 polarization and entrainment in the injury sites. AMD3100 regulate the migration of bone marrow stem cells via controlling CXCR4 binding with SDF-1. Additionally, macrophages increase the expression of hepatocyte growth factor (HGF), which is essential for regeneration, cellular growth, and movement, while also encouraging CD133^+^ stem cells to migrate into injured tissues. CD133^+^ stem cells not only promote wound re-mesothelialization, but also reduce the inflammatory response. Eventually, the burden of adhesion obviously reduces with the combination treatment after intra-abdominal surgery.

HuoXueTongFu Formula (HXTF), a traditional Chinese herbal formula, consisting of six crude herbs (including radish seed, Batsch, safflower, Natrii Sulfas, Corydalis yanhusuo, and Rheum officinale Baill), also has the function of inhibit the formation of adhesion. HXTF promote M2c macrophage polarization by activating the MerTK/PI3K/AKT signaling pathway. HXTF treatment leads to MerTK phosphorylation, which is able to activate the PI3K/Akt signaling pathway. The MerTK/PI3K/AKT signaling pathway can significantly enhance the phagocyte of macrophage, hence M2c macrophages effectively remove the neutrophil extracellular traps and reduce the inflammation in the injury sites (134, 135).

YC-1, a small molecule prototype inhibitor of hypoxia-inducible transcription factor (HIF), markedly reduces the development of postoperative adhesions by dampening HIF-1α induced M1 macrophage polarization, decreasing the recruitment and activation of peritoneal fibroblasts, alleviating EMT, and potentially promoting fibrinolysis while hindering angiogenesis (136). R243 was shown to decrease the formation of peritoneal adhesions by blocking the interaction between CCL1 and CCR8, which also disrupts the migration of macrophages into the peritoneum (137).

### 6.2 Physical barrier against macrophages

As the most widely used method in clinical, physical barrier can simply and effectively shield injury tissues to block the interconnection of adjacent abnormal tissues and avoid the adhesion formation. Barrier materials, such as hydrogels, sponge, electrospinning film, microparticle, casting film oppose diverse characteristics, mechanisms and suitable application directions. Among various materials, injectable hydrogels are applied wider for preventing adhesion and are suitable for various types of wounds owing to their high biocompatibility, ease of preparation, capability to load drugs for controlled release, flexible nature and effective reduction tissue adhesion (137). Recent research indicates that multiple kinds of hydrogel barriers exhibit anti-adhesion properties by regulating peritoneal macrophages, involving different mechanisms that contribute to this effect.

#### 6.2.1 Inhibition of macrophages accumulation

Recently, a study designed a self-leveling transient unilateral adhesive hydrogel: borate-diol complexed methacrylate gellan gum hydrogel (BMeGG-H), which presented 100% anti-adhesion effective rate (138). In a standardized intra-abdominal postoperative rat model, BMeGG-H significantly decreased the total number of F4/80^+^macrophages via below mechanisms: 1. GG is a natural polyanionic polysaccharide with excellent biocompatibility that can capture primitive macrophages by neutralizing scavenger receptors as a means of anion trapping (139). 2. The consistent barrier functions of BMeGG-H promote the repair of intestinal integrity, regulate the balance of fibrinolysis, and lead to beneficial immune modulation. The decrease in M1 macrophages resulted in the reduction of inflammatory responses, while the decrease in M2 macrophages prevented excessive ECM accumulation (138).

Another hydrogel barrier, termed sHA-ADH/OHA-E, was constructed with sulfonated HA, ADH, epigallocatechin-3-gallate (EGCG) and ROS-cleavable boronate bonds (OHA-E). Sulfonic acid groups equipped the hydrogel with commendable resistance to fibroblast and macrophage attachment, and it was demonstrated for the first time that they could serve as a strong polyanion trap to stop the clumping of free-floating GATA6^+^ macrophages via binding to scavenger receptors to prevent the formation of macrophage aggregates in a rat model of sidewall defect–cecum abrasion (18, 140). Indeed, HA is a widely used polyanionic ligand which can neutralize scavenger receptors on LPMs and inhibit the aggregation of them (141). For example, using photocurable catechol-grafted hyaluronic acid, a team designed a kind of asymmetric-adhesive hydrogel for minimally invasive surgery to prevent the formation of postoperative adhesions (141). Similarly, a study developed a sprayable, anti-inflammatory adhesion barrier, called sHAChiF. Its main functional component is also sHA (142).

The CGM hydrogel is constructed with free-radical polymerization of methacrylate chondroitin sulfate (CS-GMA) and 2-methacryloyloxyethyl phosphorylcholine (MPC) monomers. In the rat abdominal adhesion model, CGM hydrogel is able to significantly reduce the expression of CCL2 and CCR2, which are essential for the recruitment of moMacs, fibroblasts and monocytes (143). Hence, immunofluorescence staining shows that the expression of CD68 and vimentin are greatly reduced.

Using an adhesive layer (HGO) and an antiadhesive layer (CGC) to construct a Janus asymmetric-adhesive hydrogel patches (HGO-C), which is able to reduce the number of M1 macrophages and increase the number of M2 macrophages. Of note, HGO opposes excellent hemostatic property via facilitating coagulation by abundant negatively charged natural polysaccharides, exceptional adhesion capabilities and resistance to burst pressure. On the contrary, a study developed a kind of hydrogel barrier, called PCHgel, which was composited with carboxymethyl chitosan (CMCS), low Poloxamer338 (P338) and heparin. CMCS performs dual functions: it supports the creation of stable adhesion and simultaneously facilitates the controlled release of heparin. Heparin is a spectral anticoagulant drug, which has been confirmed that in the peritoneal mesothelium injury model, heparin can effectively inhibit the aggregation of peritoneal macrophages. Immunohistochemical stains shows that PCHgel significantly ameliorated the aggregation of macrophages at damaged sites, and heparin contributed to this phenomenon (21, 144).

#### 6.2.2 Regulations of macrophages phenotype

BMeGG-H significantly decreased the expression of P65 and Smad3 but increased the expression of Smad7 (145). Therefore, BMeGG-H was pivotal in promoting M2 macrophage polarization, aiding in wound healing, and decreasing fibrous deposition by regulating the TNF-α/P65 and TGF-β1/Smad signaling pathways (138). Similarly, compared with the model groups, PCHgel significantly decreased the expression of TNF-α and TGF-β1. Furthermore, PCHgel could inhibit P65 protein to block NF-κB signal pathway, hence effectively alleviating inflammatory responses and avoiding adhesion formation (144).

Similarly, A team developed a hydrogel barrier, termed AHBC/PSC, which was synthesized by phenylboronic acid (PBA)-modified hyaluronic acid (HA), adipic dihydrazide (ADH), PBA-based chlorogenic acid (CGA), polyvinyl alcohol, sulfated betaine, and p-hydroxybenzaldehyde. CGA, a natural antioxidant and anti-inflammatory drug, released by AHBC/PSC, promoted macrophages to shift from a pro-inflammatory M1 phenotype to an anti-inflammatory M2 phenotype, lead to decreased production of pro-inflammatory factors, such as iNOS, TNF-α, IL-6, IL-1β and enhanced generation of anti-inflammatory factors, such as Arg1, IL-10, CD206, thereby altering the inflammatory landscape in the abdominal cavity. Furthermore, AHBC/PSC enhanced the expression of TGF-β3 and decreased that of TGF-β1, leading to a significantly higher TGF-β3/TGF-β1 ratio, which was advantageous for preventing fibrosis and reducing its progression. In addition, AHBC/PSC was able to clear ROS via borate ester bonds (146).

As for sHA-ADH/OHA-E hydrogel barrier, ROS activation could lead to the deliberate release of EGCG by it to mitigate oxidative stress and promote the conversion of pro-inflammatory M1 macrophages into an anti-inflammatory M2 phenotype (140). The HPE-PVA Hydrogels show similar anti-oxidation and anti-inflammation properties to sHA-ADH/OHA-E hydrogel barrier (147). In addition, a zwitterionic polysaccharide-based multifunctional hydrogel containing the EGCG content, termed DSHE, is developed to exert anti-oxidation, anti-inflammation, MMT inhibition functions. The lowest CD86 and the highest CD206 expression of injury sites confirmed that DSHE barrier could effectively inhibit M1 phenotype and promote M2 phenotype via alleviating immune activation and affecting the expression of related genes (148).

An injectable adhesive-antifouling bifunctional hydrogel (AAB-hydrogel) also exert the function of preventing the formation of adhesion via inhibiting M1 macrophages and promoting M2 phenotype via the gallic acid-modified chitosan (GACS) content. The GACS possesses beneficial antioxidant properties and can significantly diminish the inflammatory response in postoperative wounds while also modulating macrophage phenotype. Of note, AAB-hydrogel is able to reducing protein disposition and resisting bacteria attacking due to good antifouling properties provided by the zwitterionic groups in the T-hydrogel (149).

In summary, inhibition of macrophage aggregates, down-regulation of macrophage pro-inflammatory factors and promoting the transformation of macrophages toward an anti-inflammatory phenotype, are the most common targets of anti-PA therapy focusing on macrophages. Current pharmacological and material-based approaches for preventing PAs demonstrate limited clinical efficacy and are often associated with adverse effects. While anti-adhesion barriers (e.g., hyaluronic acid and carboxymethyl cellulose formulations) can moderately reduce adhesion formation, their therapeutic potential remains constrained by suboptimal biocompatibility, unpredictable degradation kinetics, and poor intra-abdominal retention stability (150, 151). In the future, the new targets will be available following elucidation of mechanism of macrophages in the adhesion formation (**Table 2**).

**Table 2:** Hydrogel barriers against macrophages for PA prevention.

Hydrogels	Components	Therapeutic effect	References
BMeGG-H	gellan gum (GG); methacrylic anhydride (MA); borate-diol;	1. Decrease the total number of macrophages, including both M1 and M2 macrophages. 2. Inhibit TNF-α/P65 signaling pathways to alleviate inflammation. 3. Suppress the TGF-β1/Smad3 signaling pathway while activate TGF-β1/Smad7 to reduce fibrosis. 4. shift the fibrinolytic balance toward fibrinolysis via reducing the expression of PAI-1 while increasing the expression of t-PA	(138)
sHA-ADH/OHA-E	Sulfonated hyaluronic acid (HA); adipic dihydrazide (ADH); phenylboronic acid (PBA); epigallocatechin-3-gallate (EGCG); ROS-cleavable boronate bonds (OHA-E)	1. Release EGCG according to ROS levels to promote M2 phenotype macrophage polarization and scavenge excessive ROS to alleviate oxidative stress 2. Imped uncontrolled aggregation of GATA6+ macrophage via neutralizing scavenger receptors 3. Resist fibrin deposition, fibroblast and macrophage attachment 4. Restore fibrinolytic system balance to reduce fibrosis	(140)
DSHE	Dex-derived dextran (Dex-SB); hydroxybutyl chitosan (HBC); epigallocatechin-3-gallate (EGCG)	1. Modulate the phenotype of macrophages from M1 to M2 2. Inhibit the MMT process of peritoneal mesothelial cells 3. Mitigate inflammation by reducing excessive ROS levels and inhibiting the expression of inflammatory cytokines 4. Suppress protein, fibrinogen and collagen adsorption 5. Antibacterial property via inhibiting bacterial adhesion and killing bacteria.	(148)
CGM	methacrylate chondroitin sulfate (CS-GMA); 2-methacryloyloxyethyl phosphorylcholine (MPC)	1. Downregulate the expression of CCL2 and CCR2 to reduce the recruitment and aggregation of macrophages and fibroblasts. 2. Modulate inflammation by reducing TNF-α and Increasing IL-10 3. Inhibit the release of fibrosis-related cytokines, such as PAI-1, Col I, TGF-βR1 and increase the expression of fibrinolysis-related cytokines t-PA, MMP-9 4. Resist protein, erythrocyte and fibrinogen adsorption	(143)
HAD polymer	photocurable catechol-grafted hyaluronic acid; 3,4-dihydroxyphenylalanine (DA); 2-aminoethyl methacrylate (AEMA);	1. Promote the transition of M1-to-M2 phase 2. Induce M2 polarization and promote wound healing 3. Capture LPMs as a polyanion trap 4. Reduce the release of inflammatory cytokines, such as TNF-α, TGF-β1, and IL-6	(141)
HGO-C	tris(hydroxymethyl)aminomethane (Tris); modified hyaluronic acid (HT); modified gelatin (GT); oxidized dextran (ODEX); chitosan (CS); gelatin (Gel); Carboxylated cellulose nanofibers aqueous dispersion solution (C-CNF)	1. Convert pro-inflammatory M1 macrophages to anti-inflammatory M2 macrophages. 2. Reduce the number of M1 macrophages while increase the number of M2 macrophages. 3. Promote blood vessel formation. 4. Facilitate coagulation cascades for rapid hemostasis. 5. Inhibit bacterial proliferation	(154)
HPE-PVA	epigallocatechin-3-gallate (EGCG); hyaluronic acid (HA)-based microgels; poly (vinyl alcohol) (PVA); boronic acid	1. Inhibit M1 polarization. 2. Hinder erythrocyte, fibroblasts adhesion. 3. Reduce intracellular ROS and pro-inflammatory cytokine secretions, such as iNOS, TNF-α and IL-1β. 4. Downregulate PAI-1 expressions.	(147)
PCHgel	carboxymethyl chitosan (CMCS); CaCl2; Poloxamer338 (P338); Heparin	1. Suppress macrophages aggregation. 2. Inhibit the NF-κB signal pathway via restraining the aggregation of p65 protein. 3. Anti-inflammatory effect and inhibition of fibrosis by reducing the expression level of TNF-α and TGF-β1. 4. Reduce the level of TAFI and increase the level of AT-III to promote fibrinolysis.	(144)
AHBC/PSC	phenylboronic acid (PBA)modified hyaluronic acid (HA-PBA); adipic dihydrazide (ADH); PBA-based chlorogenic acid (CGA); polyvinyl alcohol (PVA); sulfated betaine (SB); p-hydroxybenzaldehyde (–CHO)	1. Promote the polarization of macrophages from proinflammatory M1 phenotype to anti-inflammatory M2 phenotype. 2. Reshape the inflammatory microenvironment by reducing the secretion of pro- inflammatory factors, such as iNOS and increasing the production of anti- inflammatory factors, such as Arg1 and CD206. 3. Reshape the balance of the fibrinolytic system and alleviate the fibrosis process by increasing TGF-β_3_/TGF-β_1_ ratio. 4. Strong scavenging capacity for multiple ROS. 5. Reduce the protein adsorption and fibroblast adhesion.	(146)
AAB-hydrogel	gallic acid-modified chitosan (GACS); aldehyde-modified dextran (Dex-CHO); aldehyde-modified zwitterionic dextran/carboxymethyl chitosan (Dex-SB-CHO/CMCS)	1. Suppress the polarization of macrophage to M1 phenotype and promote the polarization to the M2 phenotype. 2. Modulate inflammation by reducing pro-inflammatory factors, such as the production of IL-1β while elevating the secretion of anti-inflammatory factors, such as IL-10. 3. Improve wound healing by the antioxidant activity and prompting cell adhesion ability. 4. Prevent bacterial adhesion and inhibit bacterial growth 5. Form antifouling top to avoid adhesion to surrounding tissue.	(149)
sHAChiF	sulfated hyaluronic acid (sHA); chitosan (Chi); thermoresponsive pluronic^®^ F127	1. Suppress macrophages aggregation. 2. Reduce proinflammatory cytokines, such as IL-1β, TNF-α, IL-6 3. Modulate M1-M2 macrophage polarization and promote M2 polarization 4. Inhibit the NF-κB signal pathway 5. Disperse the peritoneal macrophages	(142)

## 7 Conclusion remark

Although PAs have been discovered and studied for a century, most of the mechanisms underlying the formation of adhesions are still unclear. The prevention and treatment of PAs and related complications are still major medical problems. Peritoneal macrophages serve as sentinels of the peritoneal immune system and are crucial for maintaining peritoneal homeostasis, eliminating pathogens and repairing damage. With the application of new tracer techniques and new analysis methods, recent studies have significantly broadened our knowledge of peritoneal macrophages. To begin with, peritoneal cavity contains embryo and HSC-derived multiple macrophage subgroups. Distinct subpopulations possess distinct renewal modes, phenotypes characteristics and functional traits which are controlled by the peritoneal local microenvironment. Besides, LPMs are highly similar to coelomocytes in the coelomic cavity of invertebrates in many aspects, such as location, function and behavior. Of note, the clusters of human cavity macrophages are obviously different from the mouse, so it still remains to be further explored whether these studies translate directly to humans. Thus, peritoneal macrophages exist extensively in different species and play a crucial in the first line of immunity in the peritoneal cavity in mammals. In conclusion, clarifying the heterogeneity of macrophages and the mechanisms underlying their role in tissue repair, inflammation, coagulation, fibrosis and fibrinolysis are necessary for understanding their regulations in PAs formation. But it remains a challenge to precisely regulate the degree of inflammation, coagulation, fibrosis and fibrinolysis to prevent the formation without hindering tissue healing.

Peritoneal macrophages possess the ability to rapidly respond to various pathogens and serosal injuries. Upon activation, macrophages aggregate to encapsulate pathogens or injuries, or engulf pathogens and die, or migrate to the omentum for antigen presentation. These events will result in MDR that initiate and regulate tissue repair. However, suffering from distinct degree serosal injuries, peritoneal macrophages present disparate behaviors, forming cell aggregates or a monolayer cell barrier. In fact, cell aggregates can also serve as physical barriers. However, once cell aggregates develop into super-aggregates when monolayer cell barrier is not adequate, PAs will form in the injury area. And it’s difficult to judge whether macrophage reaction matches the range of injury or not. In addition, peritoneal macrophages play a vital role in regulating coagulation, inflammation, fibrosis and fibrinolysis, which are closely relative to PAs formation and tissue repair. In summary, peritoneal macrophages are central players in the process of adhesion formation. However, the mechanisms underlying the switch of M1-like to M2-like phenotype are still unclear, so it’s difficult to find a target to control the role of macrophages. And whether there are exclusives pro-fibrosis or anti-fibrosis macrophage subsets need to be further studied.

The optimal approach to anti-PA therapy is to prevent adhesion formation while ensuring the wound healing. Intervention in the LPMs response has shed some light on the prevention of postoperative peritoneal formation. In fact, recent studies have developed some anti-PA strategies are closely associated with peritoneal macrophages. Drugs that act on coagulation, inflammation and HIF, can reduce the formation of PAs, However, drugs can also result in some adverse response, such as bleeding, fibrosis. In clinical, physical barrier is the most widely used method. Among various materials, injectable hydrogels are applied wider owing to their advantages, such as high biocompatibility, ease of preparation, capability to load drugs for controlled release, flexible nature and effective reduction tissue adhesion. What’s more, hydrogels mainly focus on, anti-inflammation, reducing the aggregation of macrophages and promoting M2-like macrophage polarization via various mechanisms. In conclusion, further exploration of the other molecular mechanisms of adhesion formation is crucial for creating new therapeutic strategies for PAs.

## References

[1] Hellebrekers BW Kooistra T. Pathogenesis of postoperative adhesion formation. Br J Surg. (2011) 98:1503–16. doi: 10.1002/bjs.7657, PMID: 21877324

[2] Tang J Xiang Z Bernards MT Chen S. Peritoneal adhesions: Occurrence, prevention and experimental models. Acta Biomater. (2020) 116:84–104. doi: 10.1016/j.actbio.2020.08.036, PMID: 32871282

[3] Okabayashi K Ashrafian H Zacharakis E Hasegawa H Kitagawa Y Athanasiou T. Adhesions after abdominal surgery: a systematic review of the incidence, distribution and severity. Surg Today. (2014) 44:405–20. doi: 10.1007/s00595-013-0591-8, PMID: 23657643

[4] Stommel MWJ Ten Broek RPG Strik C Slooter GD Verhoef C Grunhagen DJ. Multicenter observational study of adhesion formation after open-and laparoscopic surgery for colorectal cancer. Ann Surg. (2018) 267:743–8. doi: 10.1097/SLA.0000000000002175, PMID: 28207436

[5] ten Broek RP Issa Y van Santbrink EJ Bouvy ND Kruitwagen RF Jeekel J. Burden of adhesions in abdominal and pelvic surgery: systematic review and met-analysis. BMJ. (2013) 347:f5588. doi: 10.1136/bmj.f5588, PMID: 24092941 PMC3789584

[6] Mizuta R Mizuno Y Chen X Kurihara Y Taguchi T. Evaluation of an octyl group-modified Alaska pollock gelatin-based surgical sealant for prevention of postoperative adhesion. Acta Biomater. (2021) 121:328–38. doi: 10.1016/j.actbio.2020.12.025, PMID: 33326886

[7] Fu S Yelorda K Knowlton L. Are statins associated with reduced risk of adhesion-related complications after abdominal surgery? JAMA Netw Open. (2021) 4:e2037296. doi: 10.1001/jamanetworkopen.2020.37296, PMID: 33533927

[8] Herrick SE Allen JE. Surgical adhesions: A sticky macrophage problem. Science. (2021) 371:993–4. doi: 10.1126/science.abg5416, PMID: 33674481

[9] Mariadas H Chen JH Chen KH. The molecular and cellular mechanisms of endometriosis: from basic pathophysiology to clinical implications. Int J Mol Sci. (2025) 26:2458. doi: 10.3390/ijms26062458, PMID: 40141102 PMC11941934

[10] Ellis H Moran BJ Thompson JN Parker MC Wilson MS Menzies D. Adhesion-related hospital readmissions after abdominal and pelvic surgery: a retrospective cohort study. Lancet. (9163) 1999:353. doi: 10.1016/S0140-6736(98)09337-4, PMID: 10232313

[11] Smith SA Travers RJ Morrissey JH. How it all starts: Initiation of the clotting cascade. Crit Rev Biochem Mol Biol. (2015) 50:326–36. doi: 10.3109/10409238.2015.1050550, PMID: 26018600 PMC4826570

[12] Wang Y Zhai WJ Yang L Cheng SJ Cui WG Li JH. Establishments and evaluations of post-operative adhesion animal models. Adv Ther-Germany. (2023) 6:2200297. doi: 10.1002/adtp.202200297

[13] Choi B Lee C Yu JW. Distinctive role of inflammation in tissue repair and regeneration. Arch Pharm Res. (2023) 46:78–89. doi: 10.1007/s12272-023-01428-3, PMID: 36719600

[14] Vega-Perez A Villarrubia LH Godio C Gutierrez-Gonzalez A Feo-Lucas L Ferriz M. Resident macrophage-dependent immune cell scaffolds drive anti-bacterial defense in the peritoneal cavity. Immunity. (2021) 54:2578–2594.e5. doi: 10.1016/j.immuni.2021.10.007, PMID: 34717795

[15] Greenlee-Wacker MC. Clearance of apoptotic neutrophils and resolution of inflammation. Immunol Rev. (2016) 273:357–70. doi: 10.1111/imr.12453, PMID: 27558346 PMC5000862

[16] Pakyari M Farrokhi A Maharlooei MK Ghahary A. Critical role of transforming growth factor beta in different phases of wound healing. Adv Wound Care (New Rochelle). (2013) 2:215–24. doi: 10.1089/wound.2012.0406, PMID: 24527344 PMC3857353

[17] Fischer A Wannemacher J Christ S Koopmans T Kadri S Zhao J. Neutrophils direct preexisting matrix to initiate repair in damaged tissues. Nat Immunol. (2022) 23:518–31. doi: 10.1038/s41590-022-01166-6, PMID: 35354953 PMC8986538

[18] Zindel J Peiseler M Hossain M Deppermann C Lee WY Haenni B. Primordial GATA6 macrophages function as extravascular platelets in sterile injury. Science. (2021) 371:eabe0595. doi: 10.1126/science.abe0595, PMID: 33674464

[19] Pal R Thomas CM Salamat K Jenkins SJ Bradford BM Mabbott NA. Acute LPS exposure enhances susceptibility to peripheral prion infection. Sci Rep. (2025) 15:9754. doi: 10.1038/s41598-025-94003-3, PMID: 40119036 PMC11928655

[20] Gay D Ghinatti G Guerrero-Juarez CF Ferrer RA Ferri F Lim CH. Phagocytosis of Wnt inhibitor SFRP4 by late wound macrophages drives chronic Wnt activity for fibrotic skin healing. Sci Adv. (2020) 6:eaay3704. doi: 10.1126/sciadv.aay3704, PMID: 32219160 PMC7083618

[21] Zhang N Czepielewski RS Jarjour NN Erlich EC Esaulova E Saunders BT. Expression of factor V by resident macrophages boosts host defense in the peritoneal cavity. J Exp Med. (2019) 216:1291–300. doi: 10.1084/jem.20182024, PMID: 31048328 PMC6547866

[22] Simoes FC Cahill TJ Kenyon A Gavriouchkina D Vieira JM Sun X. Macrophages directly contribute collagen to scar formation during zebrafish heart regeneration and mouse heart repair. Nat Commun. (2020) 11:600. doi: 10.1038/s41467-019-14263-2, PMID: 32001677 PMC6992796

[23] Zindel J Mittner J Bayer J April-Monn SL Kohler A Nusse Y. Intraperitoneal microbial contamination drives post-surgical peritoneal adhesions by mesothelial EGFR-signaling. Nat Commun. (2021) 12:7316. doi: 10.1038/s41467-021-27612-x, PMID: 34916513 PMC8677808

[24] Namvar S Woolf AS Zeef LA Wilm T Wilm B Herrick SE. Functional molecules in mesothelial-to-mesenchymal transition revealed by transcriptome analyses. J Pathol. (2018) 245:491–501. doi: 10.1002/path.5101, PMID: 29774544 PMC6055603

[25] Koopmans T Rinkevich Y. Mesothelial to mesenchyme transition as a major developmental and pathological player in trunk organs and their cavities. Commun Biol. (2018) 1:170. doi: 10.1038/s42003-018-0180-x, PMID: 30345394 PMC6191446

[26] Zwicky SN Stroka D Zindel J. Sterile injury repair and adhesion formation at serosal surfaces. Front Immunol. (2021) 12:684967. doi: 10.3389/fimmu.2021.684967, PMID: 34054877 PMC8160448

[27] Okabe Y. Immune niche within the peritoneal cavity. Curr Top Microbiol Immunol. (2021) 434:123–34. doi: 10.1007/978-3-030-86016-5_6, PMID: 34850285

[28] Isaza-Restrepo A Martin-Saavedra JS Velez-Leal JL Vargas-Barato F Riveros-Duenas R. The peritoneum: beyond the tissue - A review. Front Physiol. (2018) 9:738. doi: 10.3389/fphys.2018.00738, PMID: 29962968 PMC6014125

[29] Festing MF Legg R Eydmann T Brammall A. Mouse strain differences in resident peritoneal cells: a flow cytometric analysis. Lab Anim. (1990) 24:53–62. doi: 10.1258/002367790780890374, PMID: 2304327

[30] Gazvani R Templeton A. Peritoneal environment, cytokines and angiogenesis in the pathophysiology of endometriosis. Reproduction. (2002) 123:217–26. doi: 10.1530/rep.0.1230217, PMID: 11866688

[31] Liu M Silva-Sanchez A Randall TD Meza-Perez S. Specialized immune responses in the peritoneal cavity and omentum. J Leukoc Biol. (2021) 109:717–29. doi: 10.1002/JLB.5MIR0720-271RR, PMID: 32881077 PMC7921210

[32] Bain CC Jenkins SJ. The biology of serous cavity macrophages. Cell Immunol. (2018) 330:126–35. doi: 10.1016/j.cellimm.2018.01.003, PMID: 29397065

[33] Cassado Ados A D’Imperio Lima MR Bortoluci KR. Revisiting mouse peritoneal macrophages: heterogeneity, development, and function. Front Immunol. (2015) 6:225. doi: 10.3389/fimmu.2015.00225, PMID: 26042120 PMC4437037

[34] Hashimoto D Chow A Noizat C Teo P Beasley MB Leboeuf M. Tissue-resident macrophages self-maintain locally throughout adult life with minimal contribution from circulating monocytes. Immunity. (2013) 38:792–804. doi: 10.1016/j.immuni.2013.04.004, PMID: 23601688 PMC3853406

[35] Lazarov T Juarez-Carreno S Cox N Geissmann F. Physiology and diseases of tissue-resident macrophages. Nature. (2023) 618:698–707. doi: 10.1038/s41586-023-06002-x, PMID: 37344646 PMC10649266

[36] Bain CC Hawley CA Garner H Scott CL Schridde A Steers NJ. Long-lived self-renewing bone marrow-derived macrophages displace embryo-derived cells to inhabit adult serous cavities. Nat Commun. (2016) 7:ncomms11852. doi: 10.1038/ncomms11852, PMID: 27292029 PMC4910019

[37] Ginhoux F Guilliams M. Tissue-resident macrophage ontogeny and homeostasis. Immunity. (2016) 44:439–49. doi: 10.1016/j.immuni.2016.02.024, PMID: 26982352

[38] Sahputra R Dejyong K Woolf AS Mack M Allen JE Ruckerl D. Monocyte-derived peritoneal macrophages protect C57BL/6 mice against surgery-induced adhesions. Front Immunol. (2022) 13:1000491. doi: 10.3389/fimmu.2022.1000491, PMID: 36275765 PMC9583908

[39] Jayakumar P Laganson A Deng M. GATA6(+) peritoneal resident macrophage: the immune custodian in the peritoneal cavity. Front Pharmacol. (2022) 13:866993. doi: 10.3389/fphar.2022.866993, PMID: 35401237 PMC8984154

[40] Ghosn EE Cassado AA Govoni GR Fukuhara T Yang Y Monack DM. Two physically, functionally, and developmentally distinct peritoneal macrophage subsets. Proc Natl Acad Sci U S A. (2010) 107:2568–73. doi: 10.1073/pnas.0915000107, PMID: 20133793 PMC2823920

[41] Dick SA Wong A Hamidzada H Nejat S Nechanitzky R Vohra S. Three tissue resident macrophage subsets coexist across organs with conserved origins and life cycles. Sci Immunol. (2022) 7:eabf7777. doi: 10.1126/sciimmunol.abf7777, PMID: 34995099

[42] Chakarov S Lim HY Tan L Lim SY See P Lum J. Two distinct interstitial macrophage populations coexist across tissues in specific subtissular niches. Science. (2019) 363:eaau0964. doi: 10.1126/science.aau0964, PMID: 30872492

[43] Gallerand A Han J Mintz RL Chen J Lee DD Chan MM. Tracing LYVE1(+) peritoneal fluid macrophages unveils two paths to resident macrophage repopulation with differing reliance on monocytes. bioRxiv. (2025). doi: 10.1101/2025.03.19.644175, PMID: 40166277 PMC11957119

[44] Yona S Kim KW Wolf Y Mildner A Varol D Breker M. Fate mapping reveals origins and dynamics of monocytes and tissue macrophages under homeostasis. Immunity. (2013) 38:79–91. doi: 10.1016/j.immuni.2012.12.001, PMID: 23273845 PMC3908543

[45] Bain CC Gibson DA Steers NJ Boufea K Louwe PA Doherty C. Rate of replenishment and microenvironment contribute to the sexually dimorphic phenotype and function of peritoneal macrophages. Sci Immunol. (2020) 5:eabc4466. doi: 10.1126/sciimmunol.abc4466, PMID: 32561560 PMC7610697

[46] Hoyer FF Naxerova K Schloss MJ Hulsmans M Nair AV Dutta P. Tissue-specific macrophage responses to remote injury impact the outcome of subsequent local immune challenge. Immunity. (2019) 51:899–914.e7. doi: 10.1016/j.immuni.2019.10.010, PMID: 31732166 PMC6892583

[47] Okabe Y Medzhitov R. Tissue-specific signals control reversible program of localization and functional polarization of macrophages. Cell. (2014) 157:832–44. doi: 10.1016/j.cell.2014.04.016, PMID: 24792964 PMC4137874

[48] Okabe Y. Molecular control of the identity of tissue-resident macrophages. Int Immunol. (2018) 30:485–91. doi: 10.1093/intimm/dxy019, PMID: 30371831

[49] Gautier EL Shay T Miller J Greter M Jakubzick C Ivanov S. Gene-expression profiles and transcriptional regulatory pathways that underlie the identity and diversity of mouse tissue macrophages. Nat Immunol. (2012) 13:1118–28. doi: 10.1038/ni.2419, PMID: 23023392 PMC3558276

[50] Gautier EL Ivanov S Lesnik P Randolph GJ. Local apoptosis mediates clearance of macrophages from resolving inflammation in mice. Blood. (2013) 122:2714–22. doi: 10.1182/blood-2013-01-478206, PMID: 23974197 PMC3795463

[51] Roberts AW Lee BL Deguine J John S Shlomchik MJ Barton GM. Tissue-resident macrophages are locally programmed for silent clearance of apoptotic cells. Immunity. (2017) 47:913–927.e6. doi: 10.1016/j.immuni.2017.10.006, PMID: 29150239 PMC5728676

[52] Louis C Cook AD Lacey D Fleetwood AJ Vlahos R Anderson GP. Specific contributions of CSF-1 and GM-CSF to the dynamics of the mononuclear phagocyte system. J Immunol. (2015) 195:134–44. doi: 10.4049/jimmunol.1500369, PMID: 26019271

[53] Satpathy AT Kc W Albring JC Edelson BT Kretzer NM Bhattacharya D. Zbtb46 expression distinguishes classical dendritic cells and their committed progenitors from other immune lineages. J Exp Med. (2012) 209:1135–52. doi: 10.1084/jem.20120030, PMID: 22615127 PMC3371733

[54] Meredith MM Liu K Darrasse-Jeze G Kamphorst AO Schreiber HA Guermonprez P. Expression of the zinc finger transcription factor zDC (Zbtb46, Btbd4) defines the classical dendritic cell lineage. J Exp Med. (2012) 209:1153–65. doi: 10.1084/jem.20112675, PMID: 22615130 PMC3371731

[55] Finlay CM Parkinson JE Zhang L Chan BHK Ajendra J Chenery A. T helper 2 cells control monocyte to tissue-resident macrophage differentiation during nematode infection of the pleural cavity. Immunity. (2023) 56:1064–1081.e10. doi: 10.1016/j.immuni.2023.02.016, PMID: 36948193 PMC7616141

[56] Rosas M Davies LC Giles PJ Liao CT Kharfan B Stone TC. The transcription factor Gata6 links tissue macrophage phenotype and proliferative renewal. Science. (2014) 344:645–8. doi: 10.1126/science.1251414, PMID: 24762537 PMC4185421

[57] Cain DW O’Koren EG Kan MJ Womble M Sempowski GD Hopper K. Identification of a tissue-specific, C/EBPbeta-dependent pathway of differentiation for murine peritoneal macrophages. J Immunol. (2013) 191:4665–75. doi: 10.4049/jimmunol.1300581, PMID: 24078688 PMC3808250

[58] Kim KW Williams JW Wang YT Ivanov S Gilfillan S Colonna M. MHC II+ resident peritoneal and pleural macrophages rely on IRF4 for development from circulating monocytes. J Exp Med. (2016) 213:1951–9. doi: 10.1084/jem.20160486, PMID: 27551152 PMC5030807

[59] Gundra UM Girgis NM Gonzalez MA San Tang M Van Der Zande HJP Lin JD. Vitamin A mediates conversion of monocyte-derived macrophages into tissue-resident macrophages during alternative activation. Nat Immunol. (2017) 18:642–53. doi: 10.1038/ni.3734, PMID: 28436955 PMC5475284

[60] Buechler MB Kim KW Onufer EJ Williams JW Little CC Dominguez CX. A stromal niche defined by expression of the transcription factor WT1 mediates programming and homeostasis of cavity-resident macrophages. Immunity. (2019) 51:119–130.e5. doi: 10.1016/j.immuni.2019.05.010, PMID: 31231034 PMC6814267

[61] Lavin Y Winter D Blecher-Gonen R David E Keren-Shaul H Merad M. Tissue-resident macrophage enhancer landscapes are shaped by the local microenvironment. Cell. (2014) 159:1312–26. doi: 10.1016/j.cell.2014.11.018, PMID: 25480296 PMC4437213

[62] Human and mouse peritoneal macrophages and dendritic cells compared. Nat Immunol. (2024) 25:17–8. doi: 10.1038/s41590-023-01709-5, PMID: 38168962

[63] Ardavin C Alvarez-Ladron N Ferriz M Gutierrez-Gonzalez A Vega-Perez A. Mouse tissue-resident peritoneal macrophages in homeostasis, repair, infection, and tumor metastasis. Adv Sci (Weinh). (2023) 10:e2206617. doi: 10.1002/advs.202206617, PMID: 36658699 PMC10104642

[64] Arandjelovic S Ravichandran KS. Phagocytosis of apoptotic cells in homeostasis. Nat Immunol. (2015) 16:907–17. doi: 10.1038/ni.3253, PMID: 26287597 PMC4826466

[65] Fond AM Ravichandran KS. Clearance of dying cells by phagocytes: mechanisms and implications for disease pathogenesis. Adv Exp Med Biol. (2016) 930:25–49. doi: 10.1007/978-3-319-39406-0_2, PMID: 27558816 PMC6721615

[66] Ansel KM Harris RB Cyster JG. CXCL13 is required for B1 cell homing, natural antibody production, and body cavity immunity. Immunity. Jan. (2002) 16:67–76. doi: 10.1016/s1074-7613(01)00257-6, PMID: 11825566

[67] Baumgarth N. B-1 cell heterogeneity and the regulation of natural and antigen-induced IgM production. Front Immunol. (2016) 7:324. doi: 10.3389/fimmu.2016.00324, PMID: 27667991 PMC5016532

[68] Das A Sinha M Datta S Abas M Chaffee S Sen CK. Monocyte and macrophage plasticity in tissue repair and regeneration. Am J Pathol Oct. (2015) 185:2596–606. doi: 10.1016/j.ajpath.2015.06.001, PMID: 26118749 PMC4607753

[69] Ruiz-Alcaraz AJ Carmona-Martinez V Tristan-Manzano M Machado-Linde F Sanchez-Ferrer ML Garcia-Penarrubia P. Characterization of human peritoneal monocyte/macrophage subsets in homeostasis: Phenotype, GATA6, phagocytic/oxidative activities and cytokines expression. Sci Rep. (2018) 8:12794. doi: 10.1038/s41598-018-30787-x, PMID: 30143680 PMC6109155

[70] Zindel J Kubes P. DAMPs, PAMPs, and LAMPs in immunity and sterile inflammation. Annu Rev Pathol. (2020) 15:493–518. doi: 10.1146/annurev-pathmechdis-012419-032847, PMID: 31675482

[71] Burk AM Martin M Flierl MA Rittirsch D Helm M Lampl L. Early complementopathy after multiple injuries in humans. Shock. (2012) 37:348–54. doi: 10.1097/SHK.0b013e3182471795, PMID: 22258234 PMC3306539

[72] Paidassi H Tacnet-Delorme P Garlatti V Darnault C Ghebrehiwet B Gaboriaud C. C1q binds phosphatidylserine and likely acts as a multiligand-bridging molecule in apoptotic cell recognition. J Immunol. (2008) 180:2329–38. doi: 10.4049/jimmunol.180.4.2329, PMID: 18250442 PMC2632962

[73] Beamer E Fischer W Engel T. The ATP-gated P2X7 receptor as a target for the treatment of drug-resistant epilepsy. Front Neurosci. (2017) 11:21. doi: 10.3389/fnins.2017.00021, PMID: 28210205 PMC5288361

[74] Honda M Kadohisa M Yoshii D Komohara Y Hibi T. Directly recruited GATA6 + peritoneal cavity macrophages contribute to the repair of intestinal serosal injury. Nat Commun. (2021) 12:7294. doi: 10.1038/s41467-021-27614-9, PMID: 34911964 PMC8674319

[75] Jiang Y Gong Q Huang J Gong Y Tang Q Wei D. ADAM-10 regulates MMP-12 during lipopolysaccharide-induced inflammatory response in macrophages. J Immunol Res. (2022) 2022:3012218. doi: 10.1155/2022/3012218, PMID: 36157882 PMC9507754

[76] Ito T Shintani Y Fields L Shiraishi M Podaru MN Kainuma S. Cell barrier function of resident peritoneal macrophages in post-operative adhesions. Nat Commun. (2021) 12:2232. doi: 10.1038/s41467-021-22536-y, PMID: 33854051 PMC8046819

[77] Barth MW Hendrzak JA Melnicoff MJ Morahan PS. Review of the macrophage disappearance reaction. J Leukoc Biol. (1995) 57:361–7. doi: 10.1002/jlb.57.3.361, PMID: 7884305

[78] Nelson DS. Reaction to antigens *in vivo* of the peritoneal macrophages of Guinea-pigs with delayed type hypersensitivity. Effects anticoagulants other Drugs Lancet. (1963) 2:175–6. doi: 10.1016/s0140-6736(63)92808-3, PMID: 13938029

[79] Cassado Ados A de Albuquerque JA Sardinha LR Buzzo Cde L Faustino L Nascimento R. Cellular renewal and improvement of local cell effector activity in peritoneal cavity in response to infectious stimuli. PloS One. (2011) 6:e22141. doi: 10.1371/journal.pone.0022141, PMID: 21799778 PMC3142143

[80] Meza-Perez S Randall TD. Immunological functions of the omentum. Trends Immunol. (2017) 38:526–36. doi: 10.1016/j.it.2017.03.002, PMID: 28579319 PMC5812451

[81] Leak LV. Interaction of mesothelium to intraperitoneal stimulation. I. Aggregation of peritoneal cells. Lab Invest. (1983) 48:479–91. 6834788

[82] Nelson DS North RJ. The Fate of Peritoneal Macrophages after the Injection of Antigen into Guinea Pigs with Delayed-Type Hypersensitivity. Lab Invest. Jan. (1965) 14:89–101., PMID: 14258107

[83] Wang J Kubes P. A reservoir of mature cavity macrophages that can rapidly invade visceral organs to affect tissue repair. Cell. (2016) 165:668–78. doi: 10.1016/j.cell.2016.03.009, PMID: 27062926

[84] Jorch SK Surewaard BG Hossain M Peiseler M Deppermann C Deng J. Peritoneal GATA6+ macrophages function as a portal for Staphylococcus aureus dissemination. J Clin Invest. (2019) 129:4643–56. doi: 10.1172/JCI127286, PMID: 31545300 PMC6819137

[85] Wei X Xie F Zhou X Wu Y Yan H Liu T. Role of pyroptosis in inflammation and cancer. Cell Mol Immunol. (2022) 19:971–92. doi: 10.1038/s41423-022-00905-x, PMID: 35970871 PMC9376585

[86] Kayagaki N Stowe IB Lee BL O'Rourke K Anderson K Warming S. Caspase-11 cleaves gasdermin D for non-canonical inflammasome signalling. Nature. (2015) 526:666–71. doi: 10.1038/nature15541, PMID: 26375259

[87] Shi J Zhao Y Wang K Shi X Wang Y Huang H. Cleavage of GSDMD by inflammatory caspases determines pyroptotic cell death. Nature. (2015) 526:660–5. doi: 10.1038/nature15514, PMID: 26375003

[88] Lamkanfi M Dixit VM. Mechanisms and functions of inflammasomes. Cell. (2014) 157:1013–22. doi: 10.1016/j.cell.2014.04.007, PMID: 24855941

[89] Man SM Karki R Kanneganti TD. Molecular mechanisms and functions of pyroptosis, inflammatory caspases and inflammasomes in infectious diseases. Immunol Rev. (2017) 277:61–75. doi: 10.1111/imr.12534, PMID: 28462526 PMC5416822

[90] Lu F Lan Z Xin Z He C Guo Z Xia X. Emerging insights into molecular mechanisms underlying pyroptosis and functions of inflammasomes in diseases. J Cell Physiol. (2020) 235:3207–21. doi: 10.1002/jcp.29268, PMID: 31621910 PMC13482838

[91] Song H Yang B Li Y Qian A Kang Y Shan X. Focus on the mechanisms and functions of pyroptosis, inflammasomes, and inflammatory caspases in infectious diseases. Oxid Med Cell Longev. (2022) 2022:2501279. doi: 10.1155/2022/2501279, PMID: 35132346 PMC8817853

[92] Liu Z Gu Y Chakarov S Bleriot C Kwok I Chen X. Fate mapping via Ms4a3-expression history traces monocyte-derived cells. Cell. (2019) 178:1509–1525.e19. doi: 10.1016/j.cell.2019.08.009, PMID: 31491389

[93] Louwe PA Badiola Gomez L Webster H Perona-Wright G Bain CC Forbes SJ. Recruited macrophages that colonize the post-inflammatory peritoneal niche convert into functionally divergent resident cells. Nat Commun. (2021) 12:1770. doi: 10.1038/s41467-021-21778-0, PMID: 33741914 PMC7979918

[94] Guilliams M Scott CL. Does niche competition determine the origin of tissue-resident macrophages? Nat Rev Immunol. (2017) 17:451–60. doi: 10.1038/nri.2017.42, PMID: 28461703

[95] Louwe PA Forbes SJ Benezech C Pridans C Jenkins SJ. Cell origin and niche availability dictate the capacity of peritoneal macrophages to colonize the cavity and omentum. Immunology. (2022) 166:458–74. doi: 10.1111/imm.13483, PMID: 35437746 PMC7613338

[96] Salm L Shim R Noskovicova N Kubes P. Gata6(+) large peritoneal macrophages: an evolutionarily conserved sentinel and effector system for infection and injury. Trends Immunol. (2023) 44:129–45. doi: 10.1016/j.it.2022.12.002, PMID: 36623953

[97] Buchmann K. Evolution of innate immunity: clues from invertebrates via fish to mammals. Front Immunol. (2014) 5:459. doi: 10.3389/fimmu.2014.00459, PMID: 25295041 PMC4172062

[98] Coffaro KA Hinegardner RT. Immune response in the sea urchin Lytechinus pictus. Science. (1977) 197:1389–90. doi: 10.1126/science.331476, PMID: 331476

[99] Smith LC Chang L Britten RJ Davidson EH. Sea urchin genes expressed in activated coelomocytes are identified by expressed sequence tags. Complement homologues and other putative immune response genes suggest immune system homology within the deuterostomes. J Immunol. (1996) 156:593–602. doi: 10.4049/jimmunol.156.2.593, PMID: 8543810

[100] Pancer Z. Dynamic expression of multiple scavenger receptor cysteine-rich genes in coelomocytes of the purple sea urchin. Proc Natl Acad Sci U S A. (2000) 97:13156–61. doi: 10.1073/pnas.230096397, PMID: 11069281 PMC27194

[101] Uderhardt S Martins AJ Tsang JS Lammermann T Germain RN. Resident macrophages cloak tissue microlesions to prevent neutrophil-driven inflammatory damage. Cell. (2019) 177:541–555.e17. doi: 10.1016/j.cell.2019.02.028, PMID: 30955887 PMC6474841

[102] Lammermann T Afonso PV Angermann BR Wang JM Kastenmuller W Parent CA. Neutrophil swarms require LTB4 and integrins at sites of cell death *in vivo* . Nature. (2013) 498:371–5. doi: 10.1038/nature12175, PMID: 23708969 PMC3879961

[103] Afonso PV Janka-Junttila M Lee YJ McCann CP Oliver CM Aamer KA. LTB4 is a signal-relay molecule during neutrophil chemotaxis. Dev Cell. (2012) 22:1079–91. doi: 10.1016/j.devcel.2012.02.003, PMID: 22542839 PMC4141281

[104] Deniset JF Belke D Lee WY Jorch SK Deppermann C Hassanabad AF. Gata6(+) pericardial cavity macrophages relocate to the injured heart and prevent cardiac fibrosis. Immunity. (2019) 51:131–140.e5. doi: 10.1016/j.immuni.2019.06.010, PMID: 31315031 PMC7574643

[105] Bowdish DM Gordon S. Conserved domains of the class A scavenger receptors: evolution and function. Immunol Rev. (2009) 227:19–31. doi: 10.1111/j.1600-065X.2008.00728.x, PMID: 19120472

[106] Buckenmaier CC 3rd Pusateri AE Harris RA Hetz SP. Comparison of antiadhesive treatments using an objective rat model. Am Surg Mar. (1999) 65:274–82. doi: 10.1177/000313489906500320, PMID: 10075309

[107] Dal-Secco D Wang J Zeng Z Kolaczkowska E Wong CH Petri B. A dynamic spectrum of monocytes arising from the in *situ* reprogramming of CCR2+ monocytes at a site of sterile injury. J Exp Med. (2015) 212:447–56. doi: 10.1084/jem.20141539, PMID: 25800956 PMC4387291

[108] Michel CC Nanjee MN Olszewski WL Miller NE. LDL and HDL transfer rates across peripheral microvascular endothelium agree with those predicted for passive ultrafiltration in humans. J Lipid Res Jan. (2015) 56:122–8. doi: 10.1194/jlr.M055053, PMID: 25398615 PMC4274060

[109] Herter J Zarbock A. Integrin regulation during leukocyte recruitment. J Immunol May 1. (2013) 190:4451–7. doi: 10.4049/jimmunol.1203179, PMID: 23606722

[110] Martinez FO Gordon S. The M1 and M2 paradigm of macrophage activation: time for reassessment. F1000Prime Rep. (2014) 6:13. doi: 10.12703/P6-13, PMID: 24669294 PMC3944738

[111] Kratofil RM Kubes P Deniset JF. Monocyte conversion during inflammation and injury. Arterioscler Thromb Vasc Biol. (2017) 37:35–42. doi: 10.1161/ATVBAHA.116.308198, PMID: 27765768

[112] Jin H Liu K Tang J Huang X Wang H Zhang Q. Genetic fate-mapping reveals surface accumulation but not deep organ invasion of pleural and peritoneal cavity macrophages following injury. Nat Commun. (2021) 12:2863. doi: 10.1038/s41467-021-23197-7, PMID: 34001904 PMC8129080

[113] Sun H Yang TL Yang A Wang X Ginsburg D. The murine platelet and plasma factor V pools are biosynthetically distinct and sufficient for minimal hemostasis. Blood. (2003) 102:2856–61. doi: 10.1182/blood-2003-04-1225, PMID: 12855561

[114] Garlapati V Luo Q Posma J Aluia M Nguyen TS Grunz K. Macrophage-expressed coagulation factor VII promotes adverse cardiac remodeling. Circ Res. (2024) 135:841–55. doi: 10.1161/CIRCRESAHA.123.324114, PMID: 39234697

[115] Ahamed J Niessen F Kurokawa T Lee YK Bhattacharjee G Morrissey JH. Regulation of macrophage procoagulant responses by the tissue factor cytoplasmic domain in endotoxemia. Blood. (2007) 109:5251–9. doi: 10.1182/blood-2006-10-051334, PMID: 17332247 PMC1890821

[116] Nelson DS. The effects of anticoagulants and other drugs on cellular and cutaneous reactions to antigen in Guinea-pigs with delayed-type hypersensitivity. Immunology. (1965) 9:219–34., PMID: 5838197 PMC1423575

[117] Grover SP Mackman N. Tissue factor: an essential mediator of hemostasis and trigger of thrombosis. Arterioscler Thromb Vasc Biol Apr. (2018) 38:709–25. doi: 10.1161/ATVBAHA.117.309846, PMID: 29437578

[118] Pawlinski R Wang JG Owens AP 3rd Williams J Antoniak S Tencati M. Hematopoietic and nonhematopoietic cell tissue factor activates the coagulation cascade in endotoxemic mice. Blood. (2010) 116:806–14. doi: 10.1182/blood-2009-12-259267, PMID: 20410508 PMC2918334

[119] Murray PJ Allen JE Biswas SK Fisher EA Gilroy DW Goerdt S. Macrophage activation and polarization: nomenclature and experimental guidelines. Immunity. (2014) 41:14–20. doi: 10.1016/j.immuni.2014.06.008, PMID: 25035950 PMC4123412

[120] Witherel CE Abebayehu D Barker TH Spiller KL. Macrophage and fibroblast interactions in biomaterial-mediated fibrosis. Adv Healthc Mater. (2019) 8:e1801451. doi: 10.1002/adhm.201801451, PMID: 30658015 PMC6415913

[121] Orecchioni M Ghosheh Y Pramod AB Ley K. Macrophage Polarization: Different Gene Signatures in M1(LPS+) vs. Classically and M2(LPS-) vs. Alternatively Activated Macrophages. Front Immunol. (2019) 10:1084. doi: 10.3389/fimmu.2019.01084, PMID: 31178859 PMC6543837

[122] Rodriguez-Morales P Franklin RA. Macrophage phenotypes and functions: resolving inflammation and restoring homeostasis. Trends Immunol. (2023) 44:986–98. doi: 10.1016/j.it.2023.10.004, PMID: 37940394 PMC10841626

[123] Dash SP Gupta S Sarangi PP. Monocytes and macrophages: Origin, homing, differentiation, and functionality during inflammation. Heliyon. (2024) 10:e29686. doi: 10.1016/j.heliyon.2024.e29686, PMID: 38681642 PMC11046129

[124] Franklin RA. Fibroblasts and macrophages: Collaborators in tissue homeostasis. Immunol Rev. (2021) 302:86–103. doi: 10.1111/imr.12989, PMID: 34101202

[125] Haimon Z Frumer GR Kim JS Trzebanski S Haffner-Krausz R Ben-Dor S. Cognate microglia-T cell interactions shape the functional regulatory T cell pool in experimental autoimmune encephalomyelitis pathology. Nat Immunol. (2022) 23:1749–62. doi: 10.1038/s41590-022-01360-6, PMID: 36456736

[126] Ural BB Yeung ST Damani-Yokota P Devlin JC de Vries M Vera-Licona P. Identification of a nerve-associated, lung-resident interstitial macrophage subset with distinct localization and immunoregulatory properties. Sci Immunol. (2020) 5:eaax8756. doi: 10.1126/sciimmunol.aax8756, PMID: 32220976 PMC7717505

[127] Henderson NC Rieder F Wynn TA. Fibrosis: from mechanisms to medicines. Nature. (2020) 587:555–66. doi: 10.1038/s41586-020-2938-9, PMID: 33239795 PMC8034822

[128] Aran D Looney AP Liu L Wu E Fong V Hsu A. Reference-based analysis of lung single-cell sequencing reveals a transitional profibrotic macrophage. Nat Immunol. (2019) 20:163–72. doi: 10.1038/s41590-018-0276-y, PMID: 30643263 PMC6340744

[129] Fischer A Koopmans T Ramesh P Christ S Strunz M Wannemacher J. Post-surgical adhesions are triggered by calcium-dependent membrane bridges between mesothelial surfaces. Nat Commun. (2020) 11:3068. doi: 10.1038/s41467-020-16893-3, PMID: 32555155 PMC7299976

[130] Helmke A Nordlohne J Balzer MS Dong L Rong S Hiss M. CX3CL1-CX3CR1 interaction mediates macrophage-mesothelial cross talk and promotes peritoneal fibrosis. Kidney Int. (2019) 95:1405–17. doi: 10.1016/j.kint.2018.12.030, PMID: 30948201

[131] Shook BA Wasko RR Rivera-Gonzalez GC Salazar-Gatzimas E Lopez-Giraldez F Dash BC. Myofibroblast proliferation and heterogeneity are supported by macrophages during skin repair. Science. (2018) 362:eaar2971. doi: 10.1126/science.aar2971, PMID: 30467144 PMC6684198

[132] Minutti CM Modak RV Macdonald F Li F Smyth DJ Dorward DA. A macrophage-pericyte axis directs tissue restoration via amphiregulin-induced transforming growth factor beta activation. Immunity. (2019) 50:645–654.e6. doi: 10.1016/j.immuni.2019.01.008, PMID: 30770250 PMC6436929

[133] Pakshir P Alizadehgiashi M Wong B Coelho NM Chen X Gong Z. Dynamic fibroblast contractions attract remote macrophages in fibrillar collagen matrix. Nat Commun. (2019) 10:1850. doi: 10.1038/s41467-019-09709-6, PMID: 31015429 PMC6478854

[134] Zhao M Chen Y Bao X Wang Z Yuan N Jin Z. HuoXueTongFu formula induces M2c macrophages via the MerTK/PI3K/AKT pathway to eliminate NETs in intraperitoneal adhesion in mice. J Ethnopharmacol. (2024) 331:118290. doi: 10.1016/j.jep.2024.118290, PMID: 38703872

[135] Wang Y Liu XY Wang Y Zhao WX Li FD Guo PR. NOX2 inhibition stabilizes vulnerable plaques by enhancing macrophage efferocytosis via MertK/PI3K/AKT pathway. Redox Biol. (2023) 64:102763. doi: 10.1016/j.redox.2023.102763, PMID: 37354827 PMC10320254

[136] Strowitzki MJ Ritter AS Radhakrishnan P Harnoss JM Opitz VM Biller M. Pharmacological HIF-inhibition attenuates postoperative adhesion formation. Sci Rep. (2017) 7:13151. doi: 10.1038/s41598-017-13638-z, PMID: 29030625 PMC5640636

[137] Liao J Li X Fan Y. Prevention strategies of postoperative adhesion in soft tissues by applying biomaterials: Based on the mechanisms of occurrence and development of adhesions. Bioact Mater. (2023) 26:387–412. doi: 10.1016/j.bioactmat.2023.02.026, PMID: 36969107 PMC10030827

[138] Cui F Shen S Ma X Fan D. Light-operated transient unilateral adhesive hydrogel for comprehensive prevention of postoperative adhesions. Adv Sci (Weinh). (2024) 11:e2403626. doi: 10.1002/advs.202403626, PMID: 38924679 PMC11348232

[139] Osmalek T Froelich A Tasarek S. Application of gellan gum in pharmacy and medicine. Int J Pharm. (2014) 466:328–40. doi: 10.1016/j.ijpharm.2014.03.038, PMID: 24657577

[140] Huang Y Dai X Gong Y Ren L Luo Y Sun Y. ROS-responsive sprayable hydrogel as ROS scavenger and GATA6(+) macrophages trap for the prevention of postoperative abdominal adhesions. J Control Release. (2024) 369:573–90. doi: 10.1016/j.jconrel.2024.03.051, PMID: 38554773

[141] Wu XQ Guo WH Wang L Xu YC Wang ZH Yang Y. An injectable asymmetric-adhesive hydrogel as a GATA6 cavity macrophage trap to prevent the formation of postoperative adhesions after minimally invasive surgery. Adv Funct Mater. (2022) 32:2110066. doi: 10.1002/adfm.202110066

[142] Song W Lee C Jeong H Kim S Hwang NS. Sprayable anti-adhesive hydrogel for peritoneal macrophage scavenging in post-surgical applications. Nat Commun. (2024) 15:8364. doi: 10.1038/s41467-024-52753-0, PMID: 39333108 PMC11436759

[143] Wen J Liu K Bu Y Zhang Y Zheng Y He J. An injectable and antifouling hydrogel prevents the development of abdominal adhesions by inhibiting the CCL2/CCR2 interaction. Biomaterials. (2024) 311:122661. doi: 10.1016/j.biomaterials.2024.122661, PMID: 38875883

[144] Lang P Liu T Huang S Zhou Z Zhang M Lin Y. Degradable temperature-sensitive hydrogel loaded with heparin effectively prevents post-operative tissue adhesions. ACS Biomater Sci Eng. (2023) 9:3618–31. doi: 10.1021/acsbiomaterials.3c00017, PMID: 37179492

[145] Yu J Wang K Fan C Zhao X Gao J Jing W. An ultrasoft self-fused supramolecular polymer hydrogel for completely preventing postoperative tissue adhesion. Adv Mater. (2021) 33:e2008395. doi: 10.1002/adma.202008395, PMID: 33734513

[146] Zhang T Huang Y Gong Y Shi X Xiao D Ren L. A ROS-responsive and scavenging hydrogel for postoperative abdominal adhesion prevention. Acta Biomater. (2024) 184:98–113. doi: 10.1016/j.actbio.2024.06.027, PMID: 38914412

[147] Liu B Kong Y Alimi OA Kuss MA Tu H Hu W. Multifunctional microgel-based cream hydrogels for postoperative abdominal adhesion prevention. ACS Nano. (2023) 17:3847–64. doi: 10.1021/acsnano.2c12104, PMID: 36779870 PMC10820954

[148] Yu Q Sun H Zhang L Jiang L Liang L Yu C. A zwitterionic hydrogel with anti-oxidative and anti-inflammatory properties for the prevention of peritoneal adhesion by inhibiting mesothelial-mesenchymal transition. Adv Healthc Mater. (2023) 12:e2301696. doi: 10.1002/adhm.202301696, PMID: 37669499

[149] Zhao Z Sun H Yu C Liu B Liu R Yang Q. Injectable asymmetric adhesive-antifouling bifunctional hydrogel for peritoneal adhesion prevention. Adv Healthc Mater. (2024) 13:e2303574. doi: 10.1002/adhm.202303574, PMID: 38115543

[150] Caglayan K Gungor B Cinar H Erdogan NY Koca B. Preventing intraperitoneal adhesions with linezolid and hyaluronic acid/carboxymethylcellulose: a comparative study in cecal abrasion model. Am J Surg. (2014) 208:106–11. doi: 10.1016/j.amjsurg.2012.05.038, PMID: 24814308

[151] Yang S Zheng Y Pu Z Nian H Li J. The multiple roles of macrophages in peritoneal adhesion. Immunol Cell Biol. (2025) 103:31–44. doi: 10.1111/imcb.12831, PMID: 39471989

[152] Barela Hudgell MA Grayfer L Smith LC. Coelomocyte populations in the sea urchin, Strongylocentrotus purpuratus, undergo dynamic changes in response to immune challenge. Front Immunol. (2022) 13:940852. doi: 10.3389/fimmu.2022.940852, PMID: 36119116 PMC9471872

[153] Coates CJ Rowley AF Smith LC Whitten MMA. 3Host defences of invertebrates to pathogens and parasites. In: Rowley AF Coates CJ Whitten MW, editors. Invertebrate Pathology. (Oxford: Oxford University Press (2022). doi: 10.1093/oso/9780198853756.003.0001

[154] Sun LF Zhou JY Lai JY Zheng X Wang HZ Lu B. Novel natural polymer-based hydrogel patches with janus asymmetric-adhesion for emergency hemostasis and wound healing. Adv Funct Mater. (2024) 34:2401030. doi: 10.1002/adfm.202401030

